# MiR-106a-5p targets PFKFB3 and improves sepsis through regulating macrophage pyroptosis and inflammatory response

**DOI:** 10.1016/j.jbc.2024.107334

**Published:** 2024-05-03

**Authors:** Yixin Chen, Ping Zhang, Fangwei Han, Yanying Zhou, Juexian Wei, Cailing Wang, Mingchuan Song, Shaopeng Lin, Yiming Xu, Xiaohui Chen

**Affiliations:** 1 Department of Emergency, the Second Affiliated Hospital, Guangzhou Medical University, Guangzhou, China; 2 School of Pharmaceutical Sciences, Guangzhou Medical University, Guangzhou, China; 3 School of Public Health, UNT Health Science Center, Fort Worth, Texas, USA; 4 School of Pharmaceutical Sciences, Sun Yat-sen University, Guangzhou, China; 5 School of Basic Medical Sciences, Guangzhou Medical University, Guangzhou, China

**Keywords:** sepsis, macrophage, PFKFB3, miRNA-106a-5p, glycolysis, inflammation, pyroptosis

## Abstract

The enzyme 6-phosphofructo-2-kinase/fructose-2,6-bisphosphatase isoform 3 (PFKFB3) is a critical regulator of glycolysis and plays a key role in modulating the inflammatory response, thereby contributing to the development of inflammatory diseases such as sepsis. Despite its importance, the development of strategies to target PFKFB3 in the context of sepsis remains challenging. In this study, we employed a miRNA-based approach to decrease PFKFB3 expression. Through multiple meta-analyses, we observed a downregulation of miR-106a-5p expression and an upregulation of PFKFB3 expression in clinical sepsis samples. These changes were also confirmed in blood monocytes from patients with early sepsis and from a mouse model of lipopolysaccharide (LPS)-induced sepsis. Overexpression of miR-106a-5p significantly decreased the LPS-induced increase in glycolytic capacity, inflammatory response, and pyroptosis in macrophages. Mechanistically, we identified PFKFB3 as a direct target protein of miR-106a-5p and demonstrated its essential role in LPS-induced pyroptosis and inflammatory response in macrophages. Furthermore, treatment with agomir-miR-106a-5p conferred a protective effect in an LPS mouse model of sepsis, but this effect was attenuated in myeloid-specific Pfkfb3 KO mice. These findings indicate that miR-106a-5p inhibits macrophage pyroptosis and inflammatory response in sepsis by regulating PFKFB3-mediated glucose metabolism, representing a potential therapeutic option for the treatment of sepsis.

Sepsis is a life-threatening clinical syndrome induced by infection or injury, leading to the disruption of immune-inflammation homeostasis and the occurrence of multiple organ failure (1, 2). It can cause widespread cell and tissue damage due to an excessive inflammatory response (3, 4, 5). Macrophages, which regulate inflammation and immune balance, can overreact during sepsis, causing severe organ damage (6, 7, 8). Modulating macrophage function to control aberrant inflammatory responses is a useful strategy for improving the survival rates of sepsis, and holds great promise as a potential early intervention in the clinical management of sepsis.

The interplay between macrophage phenotypes and cell metabolism is increasingly recognized. In the initial stages of sepsis, signals associated with pathogens redirect macrophage metabolism from oxidative phosphorylation toward glycolysis and the pentose phosphate pathway, bolstering the inflammatory response (9, 10, 11). Glycolysis, a metabolic pathway, is facilitated by several metabolic enzymes, among which 6-phosphofructo-2-kinase/fructose-2,6-biphosphatase 3 (PFKFB3) is a critical one (12, 13). PFKFB3 expedites glycolysis by regulating and sustaining the intracellular concentrations of fructose-2,6-bisphosphate, which in turn allosterically activates 6-phosphofructokinase-1, the principal rate-limiting enzyme of glycolysis (14, 15). PFKFB3 is found to be overexpressed in various cells implicated in sepsis, including macrophages, neutrophils, endothelial cells, and lung fibroblasts (13, 15, 16). A growing body of evidence suggests that an increase in PFKFB3-driven glycolysis in immune cells is intimately linked with the excessive inflammatory response observed in sepsis (14, 17). Furthermore, the pharmacological inhibition of glycolysis by targeting PFKFB3 has been associated with a decrease in mortality, positioning PFKFB3 as a promising therapeutic target for mitigating excessive inflammation in sepsis. Therefore, understanding how to regulate glycolysis reprogramming and PFKFB3 expression could provide valuable insights for improving sepsis treatments.

miRNAs, small noncoding RNAs, regulate gene expression posttranscriptionally and have become significant tools in sepsis treatment due to advancements in nucleic acid delivery systems (18, 19, 20). MiR-106a-5p, a miRNA known for its tumor-suppressive properties, has been associated with various diseases including inflammatory bowel disease, acute cerebral infarction, and liver fibrosis (21, 22, 23). It is considered a potential biomarker for diagnosing these diseases and may influence their progression and treatment. However, the role of miR-106a-5p in macrophages and its impact on sepsis immunity remains an open area of research.

In this study, we reanalyzed publicly available RNA-seq datasets and identified miR-106a-5p as the most efficient human miRNA for abrogating PFKFB3 expression and inhibiting macrophage pyroptosis and inflammatory response in sepsis. We then evaluated the therapeutic effect of miR-106a-5p against sepsis and found that treatment with agomir-miR-106a-5p, by reducing Pfkfb3 levels, was able to alleviate macrophage pyroptosis, inflammatory response, and lung injury in a lipopolysaccharide (LPS)-induced sepsis mouse model. This study proposes, for the first time, an antiseptic miRNA-based approach for targeting Pfkfb3 *in vivo*.

## Results

### The expression levels of PFKFB3 and miR-106a-5p are oppositely regulated in sepsis

**Figure 1:**
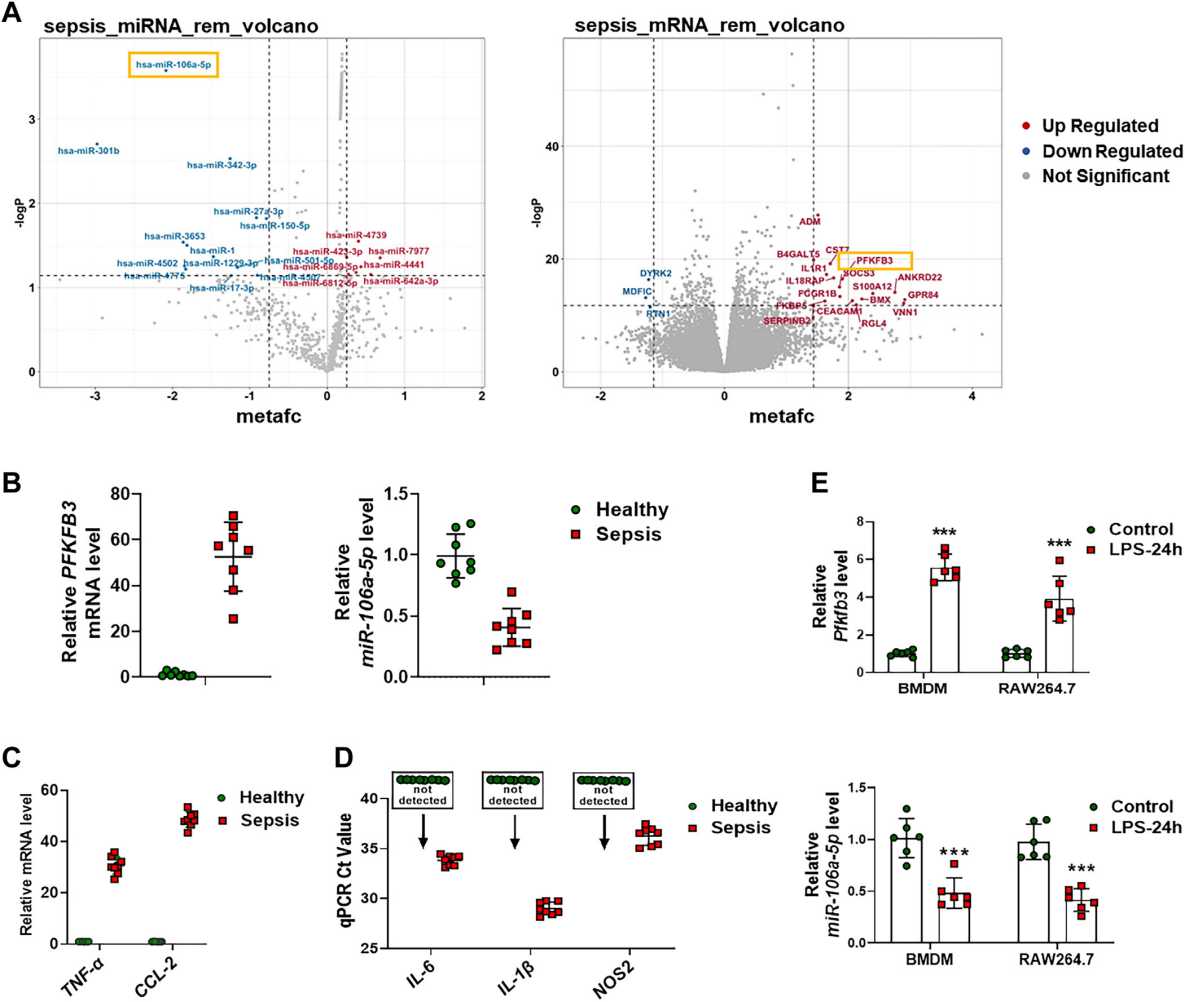
**Computational analyses and experimental verification reveal abnormal expression of PFKFB3 and miR-106a-5p in sepsis samples.***A*, the top 20 upregulated and downregulated miRNAs (*left*) or genes (*right*) in clinical sepsis samples identified through a meta-analysis using the GEO database. *B*, real-time PCR analysis of the mRNA levels of *PFKFB3* and *miR-106a-5p* in blood samples from sepsis patients or healthy controls (n = 8 per group). *C* and *D*, real-time PCR analysis of the mRNA levels of *TNF-α*, *CCL2*, *IL-6*, *IL-1β*, and *NOS2* in blood samples from sepsis patients or healthy controls (n = 8 per group). *E*, real-time PCR analysis of the mRNA levels of *Pfkfb3* (*upper*) and miR-106a-5p (*lower*) in bone marrow–derived macrophages (BMDMs) and RAW264.7 cells with 1 μg/ml LPS treatment for 24 h (n = 6 per group). Values are presented as mean ± SD, ∗∗∗*p* < 0.001 (unpaired, two-tailed Student’s *t* test). Ccl2, chemokine ligand 2; Il-1β, interleukin-1β; IL-6, interleukin-6; GEO, Gene Expression Omnibus; LPS, lipopolysaccharide; Nos2, nitric oxide synthase 2; PFKFB3, 6-phosphofructo-2-kinase/fructose-2,6-biphosphatase 3; TNF, tumor necrosis factor.

In our pursuit of an antiseptic miRNA-based strategy for targeting *PFKFB3*, we initially utilized the TARGETSCAN database to identify miRNA molecules, derived from both humans and mice, which could potentially target *PFKFB3* (Table S1). Subsequently, we conducted a meta-analysis of various types of studies using the Gene Expression Omnibus (GEO) database (Fig. S1 and Table S2). This led us to identify the top 13 miRNAs that exhibited the most significant downregulated expression changes in clinical sepsis samples, among which miR-106a-5p showed the most significant decrease (Figs. 1*A* and S2*A*). Interestingly, the meta-analysis data also revealed an increased abundance of PFKFB3 in sepsis (Figs. 1*A* and S2*B*). Comparing these 13 downregulated miRNAs with the miRNAs identified using the TARGETSCAN database; we found that miR-106a-5p was the only miRNA with the potential to target PFKFB3 in sepsis (Fig. S2*C*). Further analysis by four target prediction softwares (TARGETSCAN, https://www.targetscan.org/; MIRDB, https://mirdb.org/; PITA and MIRANDA, https://tools4mirs.org/) suggested that PFKFB3 might indeed be a potential target of miR-106a-5p.

To validate these findings, we isolated monocytes from blood samples of eight patients in the early stages of sepsis, as well as from matched controls. We observed a decrease in the mRNA level of *miR-106a-5p* in the monocytes of sepsis patients when compared to healthy controls. Conversely, the *PFKFB3* level exhibited a significant increase (Fig. 1*B*). Along with these changes, the mRNA levels of inflammatory mediators such as tumor necrosis factor-α (*Tnf-α*), chemokine ligand 2 (*Ccl2*), interleukin-6 (*Il-6*), interleukin-1β (*Il-1β*), and nitric oxide synthase 2 (*Nos2*) also demonstrated a marked elevation in the sepsis group (Fig. 1, *C* and *D*). Using a LPS-induced macrophage inflammation model, we found that LPS treatment reduced the mRNA level of *miR-106a-5p*, while simultaneously stimulated the mRNA level of *Pfkfb3* in both murine bone marrow–derived macrophages (BMDMs) and RAW264.7 cells (Fig. 1*E*). These results suggest that the levels of miR-106a-5p and PFKFB3 are oppositely regulated in septic macrophages.

### MiR-106a-5p negatively regulates Pfkfb3 in macrophages

**Figure 2:**
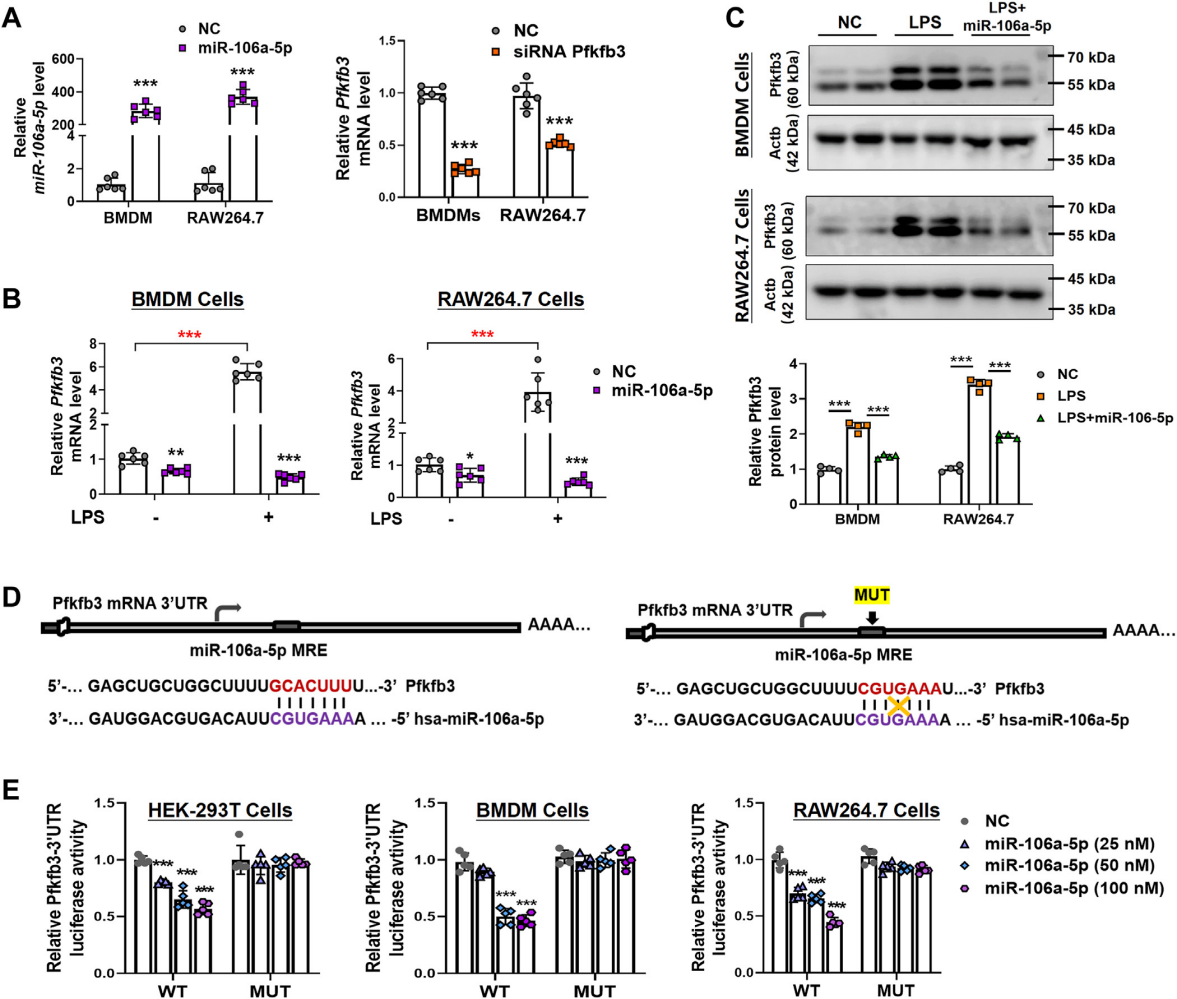
**MiR-106a-5p negatively regulates Pfkfb3.***A*, the efficiency of miR-106a-5p mimic (*left*) and si*Pfkfb3* (*right*) in BMDM and RAW264.7 cells (n = 6 per group). *B*, real-time PCR analysis of the mRNA levels of *Pfkfb3* in LPS (24 h, 1 μg/ml)-treated BMDM (*left*) and RAW264.7 (*right*) cells pretransfected with miR-106a-5p mimic (n = 6 per group). *C*, Western blot analyses of the protein levels of Pfkfb3 in LPS (24 h, 1 μg/ml)-treated BMDM (*upper*) and RAW264.7 (*lower*) cells pretransfected with miR-106a-5p mimic (n = 2 per group). The quantification of Western blot images was performed using Image J software (n = 4 biological replicates). *D*, bioinformatics analysis revealed putative MRE site for miR-106a-5p within the 3′-UTR of *Pfkfb3* transcript (*left*). The sequence of *Pfkfb3*-3′UTR bearing the mutated MRE sites (*right*) is also shown. *E*, *Pfkfb3*-3′-UTR luciferase reporter activities in HEK-293T (*left*), BMDM (*middle*), and RAW264.7 (*right*) cells with WT or mutated *Pfkfb3*-reporter plasmids (100 ng per well) pretransfected and then with 24 h miR-106a-5p mimic (2550 or 100 nM) treatment (n = 5 per group). Values are presented as mean ± SD, ∗*p* < 0.05, ∗∗*p* < 0.01, and ∗∗∗*p* < 0.001 (unpaired, two-tailed Student’s *t* test for *A*; one-way ANOVA with Tukey’s post hoc test for *B*; two-way ANOVA with Bonferroni post hoc tests for *E*). BMDM, bone marrow–derived macrophage; LPS, lipopolysaccharide; MRE, miRNA response element; PFKFB3, 6-phosphofructo-2-kinase/fructose-2,6-biphosphatase 3; *siPfkfb3*, *Pfkfb3* siRNA.

To clarify how miR-106a-5p regulates *Pfkfb3* expression in macrophages, we manipulated the expression levels of both miR-106a-5p and *Pfkfb3* using a miR-106a-5p mimic and *Pfkfb3* siRNA (si*Pfkfb3*) in BMDM and RAW264.7 cells (Fig. 2*A*). We observed that overexpression of miR-106a-5p significantly inhibited the expression of Pfkfb3 at both mRNA and protein levels in BMDMs and RAW264.7 cells (Fig. 2, *B* and *C*). Our bioinformatics analysis identified a miRNA response element within the 3′-UTR of *Pfkfb3*. To validate the target specificity of miR-106a-5p, we employed luciferase reporter assays using both the WT 3′-UTR of *Pfkfb3* and a mutant (mut) variant of *Pfkfb3*, which carries a modification at the miR-106a-5p binding site (Fig. 2*D*). Our findings revealed that miR-106a-5p significantly diminished the reporter activity of wt-*Pfkfb3* 3′-UTR in HEK-293T, RAW264.7, and BMDM cells in a dose-dependent manner (Fig. 2*E*). However, the reporter activity of mut-*Pfkfb3* 3′-UTR remained unaffected by miR-106a-5p overexpression (Fig. 2, *D* and *E*). Collectively, these results indicate that miR-106a-5p downregulates Pfkfb3 expression by directly binding to the 3′-UTR of *Pfkfb3* mRNA.

### MiR-106a-5p suppresses glycolysis in inflammatory macrophages through Pfkfb3 inhibition

**Figure 3:**
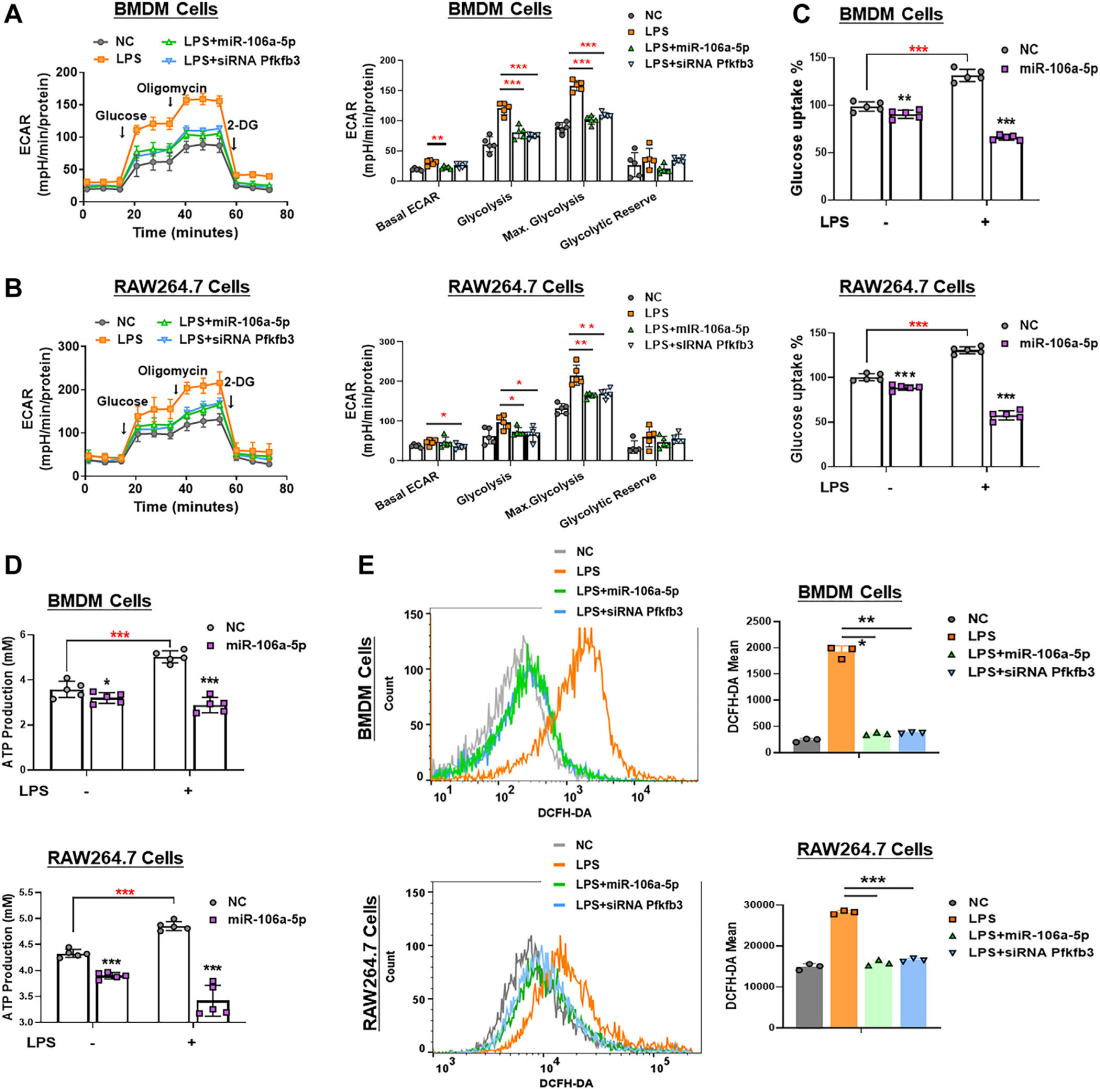
**MiR-106a-5p suppresses glycolysis in inflammatory macrophages through Pfkfb3 inhibition.***A* and *B*, ECAR profile showing glycolytic function (*left*) and quantification of glycolytic function parameters (*right*) in LPS (24 h, 1 μg/ml)-treated BMDM cells and RAW264.7 cells with miR-106a-5p mimic or si*Pfkfb3* pretransfected (n = 5 per group). *C*, glucose uptake capacity, determined by the 2-NBDG–based glucose uptake assay, in LPS (24 h, 1 μg/ml)-treated BMDM (*upper*) and RAW264.7 (*lower*) cells with miR-106a-5p mimic or si*Pfkfb3* pretransfected (n = 5 per group). *D*, the intracellular ATP content in LPS (24 h, 1 μg/ml)-treated BMDM (*upper*) and RAW264.7 (*lower*) cells with miR-106a-5p mimic or si*Pfkfb3* pretransfected (n = 5 per group). *E*, cellular ROS detection and quantification *via* DCFH-DA staining, followed by flow cytometry in LPS (24 h, 1 μg/ml)-treated BMDM (*upper*) and RAW264.7 (*lower*) cells with miR-106a-5p mimic or si*Pfkfb3* pretransfected (n = 3 per group). Values are presented as means ± SD, ∗*p* < 0.05, ∗∗*p* < 0.01, and ∗∗∗*p* < 0.001 (one-way ANOVA with Tukey’s post hoc test). BMDM, bone marrow–derived macrophage; DCFH-DA, dichlorodihydrofluorescein diacetate; ECAR, extracellular acidification rate; LPS, lipopolysaccharide; PFKFB3, 6-phosphofructo-2-kinase/fructose-2,6-biphosphatase 3; ROS, reactive oxygen species; *siPfkfb3*, *Pfkfb3* siRNA.

Accumulating evidence has demonstrated that inflammatory macrophages exhibit a high level of PFKFB3-mediated glycolysis (13, 17). Thus, we examined the effect of miR-106a-5p on the glycolysis capacity of macrophages by measuring the extracellular acidification rate (ECAR) through Seahorse Extracellular Flux analysis. Our findings revealed that LPS treatment significantly augmented glycolysis and glycolytic capacity in BMDM and RAW264.7 cells (Fig. 3, *A* and *B*). Notably, overexpression of miR-106a-5p or knockdown of *Pfkfb3* significantly reduced glycolytic metabolism in LPS-treated cells (Fig. 3, *A* and *B*). Using a 2-Deoxy-2- [(7-nitro-2,1,3-benzoxadiazol-4-yl)amino]-D-glucose–based assay, we found that transfection with either miR-106a-5p mimic or si*Pfkfb3* resulted in a significant suppression of glucose uptake in LPS-treated BMDMs and RAW264.7 cells (Fig. 3*C*). In addition, under energy stress conditions induced by serum depletion, LPS treatment markedly increased ATP production in both BMDM and RAW264.7 cells. However, this effect was negated by either miR-106a-5p mimic or si*Pfkfb3* treatment (Fig. 3*D*). Together, these findings suggest that miR-106a-5p might modulate glycolysis, which aids in extracting energy from glucose in inflammatory macrophage, through the inhibition of *Pfkfb3* expression. Interestingly, we found that overexpression of miR-106a-5p or knockdown of *Pfkfb3* also inhibited LPS-induced upregulation of mRNA levels of the glucose transporter and glycolytic enzymes, including solute carrier family 2 member 1, *Pfk1* family, and hexokinase 1 in BMDMs and RAW264.7 cells (Fig. S3*A*). This might potentially be due to a feedback regulation mechanism of metabolic inhibition by miR-106a-5p.

Considering previous studies have shown that enhanced activity of PFKFB3 accelerates reactive oxygen species (ROS) production as an end product of glycolysis in T lymphocytes (24), we conducted further investigations to detect the intracellular content of ROS in macrophages. Utilizing both dichlorodihydrofluorescein diacetate (DCFH-DA) and dihydroethidium (DHE) staining, followed by flow cytometry, we found that LPS treatment induced an increase in ROS level in BMDMs and RAW264.7 cells. Nevertheless, this effect was significantly mitigated by either miR-106a-5p mimic or si*Pfkfb3* transfection (Figs. 3*E* and S3*B*). These findings indicate that the miR-106a-5p–Pfkfb3 axis may play a crucial role in regulating ROS levels in inflammatory macrophages.

### MiR-106a-5p suppresses macrophage inflammatory response, NLRP3 inflammasome activation, and pyroptosis through Pfkfb3 inhibition

**Figure 4:**
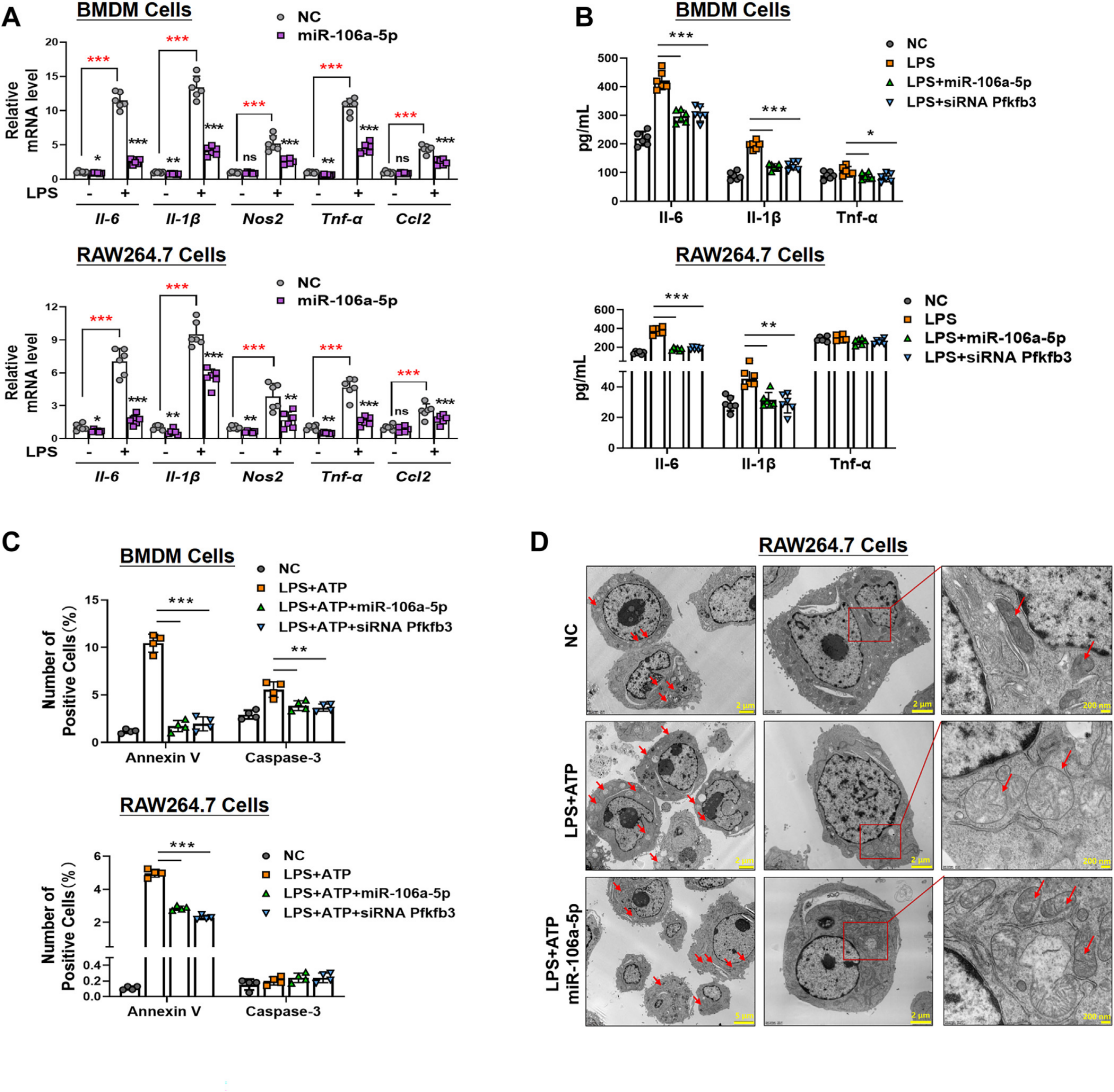
**MiR-106a-5p suppresses macrophage pyroptosis through Pfkfb3 inhibition.***A*, the mRNA levels of *Il-6*, *Il-1β*, *Nos2*, *Tnf-α*, and *Ccl2* in LPS (24 h, 1 μg/ml)-treated BMDM (*upper*) and RAW264.7 (*lower*) cells with miR-106a-5p mimic pretreatment (n = 6 per group). *B*, the Il-6, Il-1β, and Tnf-α protein levels, measured by ELISA assay, in culture medium of LPS (24 h, 1 μg/ml)-treated BMDM (*upper*) and RAW264.7 (*lower*) cells with miR-106a-5p mimic or si*PFKFB3* pretransfected (n = 6 per group). *C*, the level of apoptosis or pyroptosis determined by flow cytometry analysis of annexin V and caspase 3 in miR-106a-5p mimic or si*Pfkfb3*-transfected BMDM (*upper*) and RAW264.7 (*lower*) cells challenged with vehicle or LPS (1 μg/ml) for 24 h, followed by incubating with ATP (4 mM) for 45 min (n = 3 per group). *D*, transmission electron microscopy (TEM) showing the cell morphology in miR-106a-5p mimic-transfected RAW264.7 cells challenged with vehicle or LPS (1 μg/ml) for 24 h, followed by incubating with ATP (4 mM) for 45 min. Values are presented as means ± SD, ∗*p* < 0.05, ∗∗*p* < 0.01, and ∗∗∗*p* < 0.001 (one-way ANOVA with Tukey’s post hoc test). BMDM, bone marrow–derived macrophage; Ccl2, chemokine ligand 2; Il-1β, interleukin-1β; IL-6, interleukin-6; LPS, lipopolysaccharide; Nos2, nitric oxide synthase 2; PFKFB3, 6-phosphofructo-2-kinase/fructose-2,6-biphosphatase 3; *siPfkfb3*, *Pfkfb3* siRNA; TNF, tumor necrosis factor.

The glucose metabolism of macrophages directly influences their inflammatory responses. Therefore, we investigated the effect of miR-106a-5p on the mRNA levels of various inflammatory factors or cytokines, such as *Il-6*, *Il-1β*, *Nos2*, *Tnf-α*, and *Ccl2* in BMDMs and RAW264.7 cells under both LPS-stimulated and nonstimulated conditions. Our findings indicated that miR-106a-5p significantly inhibited the mRNA levels of these inflammatory factors in macrophages, especially those stimulated with LPS (Fig. 4*A*). Further, an ELISA revealed a notable reduction in the secretory levels of Il-6, Il-1β, and Tnf-α in the cell culture medium of BMDM and RAW264.7 cells transfected with either miR-106a-5p mimic or si*Pfkfb3* (Fig. 4*B*).

Studies have shown that the activation of the NLR family pyrin domain containing 3 (NLRP3) inflammasome and subsequent pyroptosis in macrophages can lead to the release of proinflammatory factors, such as IL-1β, thereby augmenting inflammatory responses observed in conditions like sepsis. We therefore examined whether miR-106a-5p overexpression affects macrophage pyroptosis using caspase-3-annexin V double staining and flow cytometric analyses. Our findings indicated that LPS stimulation significantly increased the number of annexin V–positive cells but had minimal effect on the expression of caspase-3 in both BMDM and RAW264.7 cells when treated with the specific NLRP3 inflammasome activator ATP (Figs. 4*C* and S4), indicating that LPS-induced inflammatory macrophages are more prone to pyroptosis than apoptosis. Interestingly, the elevated macrophage pyroptosis induced by LPS were significantly reduced by pretreatment with miR-106a-5p mimic or si*Pfkfb3* (Figs. 4C and S4). To further confirm our findings, we employed transmission electron microscopy to identify the structural changes associated with pyroptosis. Compared to the normal cellular structure observed in the control group, cell swelling and damage to multiple cellular organelles, as characterized by clouding of mitochondrial cristae and mitochondrial swelling, were observed in the group treated with LPS and ATP. However, these morphological changes induced by LPS were alleviated upon transfection with the miR-106a-5p mimic (Fig. 4*D*).

**Figure 5:**
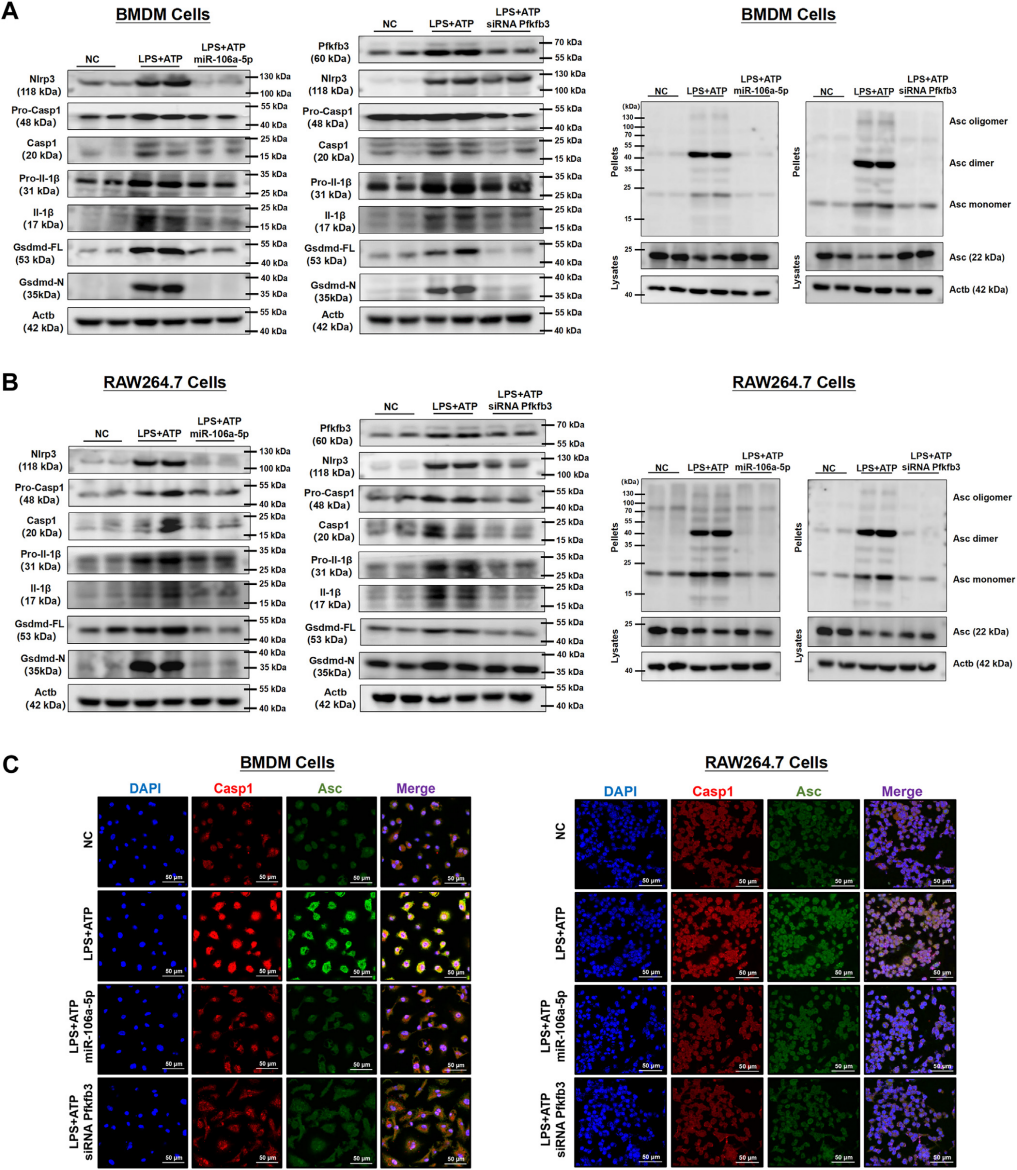
**MiR-106a-5p suppresses macrophage NLRP3 inflammasome activation through Pfkfb3 inhibition.***A* and *B*, the protein levels of Pfkfb3, Nlrp3, Pro-Casp1, cleaved Casp1, Pro-Il-1β, cleaved Il-1β, full-length Gsdmd, and N-terminal fragment of Gsdmd, as well as the level of ASC oligomerization in miR-106a-5p mimic or si*Pfkfb3*-transfected BMDM cells and RAW264.7 cells challenged with vehicle or LPS (1 μg/ml) for 24 h, followed by incubating with ATP (4 mM) for 45 min (n = 2 per group). The quantification of Western blot images was performed using Image J software (n = 4 biological replicates). *C*, IF staining of Casp-1 and Asc in miR-106a-5p mimic or si*Pfkfb3*-transfected BMDM (*left*) and RAW264.7 (*right*) cells challenged with vehicle or LPS (1 μg/ml) for 24 h, followed by incubating with ATP (4 mM) for 45 min (n = 3 per group). Asc, apoptosis-associated speck-like protein with a caspase recruitment domain; BMDM, bone marrow–derived macrophage; Ccl2, chemokine ligand 2; Gsdmd, gasdermin D; Il-1β, interleukin-1β; IL-6, interleukin-6; LPS, lipopolysaccharide; Nos2, nitric oxide synthase 2; PFKFB3, 6-phosphofructo-2-kinase/fructose-2,6-biphosphatase 3; *siPfkfb3*, *Pfkfb3* siRNA; TNF, tumor necrosis factor.

The NLRP3 inflammasome plays a crucial role in the process of pyroptosis. We therefore investigated the causal relationship between miR-106a-5p and NLRP3 inflammasome activation in macrophages. Our findings revealed that LPS treatment upregulated the expression of NLRP3 inflammasome components (Nlrp3, procaspase 1, and pro-Il-1β) and promoted the cleavage of procaspase-1, pro-IL-1β, and gasdermin D (Gsdmd), as well as the oligomerization of apoptosis-associated speck-like protein with a caspase recruitment domain (Asc) in both BMDM and RAW264.7 cells, upon specific NLRP3 inflammasome activator ATP; however, these effects were blocked by miR-106a-5p mimic or si*Pfkfb3* transfection (Figs. 5, *A* and *B* and S5). Furthermore, immunofluorescence staining revealed that LPS and ATP treatment dramatically increased the expression of Casp1 and Asc and promoted their colocalization in both BMDM and RAW264.7 cells. These effects were reversed by transfection with either miR-106a-5p mimic or si*Pfkfb3* (Fig. 5*C*).

Taken together, these results demonstrate that miR-106a-5p, potentially through the inhibition of *Pfkfb3* expression, suppress the activation of NLRP3 inflammasome and reduce the occurrence of pyroptosis in inflammatory macrophages.

### Pfkfb3 overexpression reverses the miR-106a-5p–induced suppression of inflammatory response and pyroptosis in macrophages

**Figure 6:**
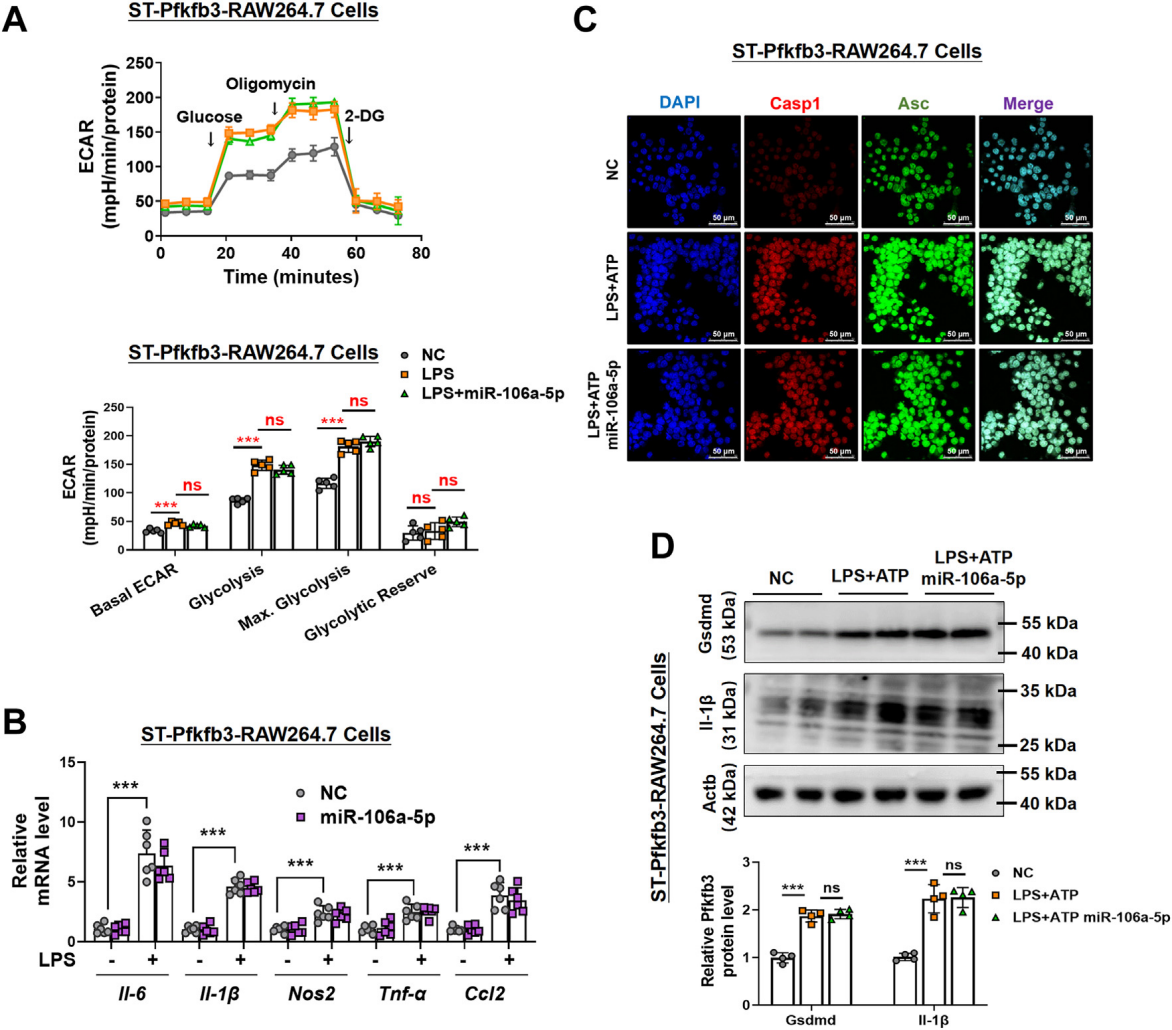
**Pfkfb3 overexpression reverses the miR-106a-5p-induced suppression of pyroptosis and inflammatory response in macrophages.***A*, ECAR profile showing glycolytic function and quantification of glycolytic function parameters in Pfkfb3 overexpressed RAW264.7 cells (ST-Pfkfb3-RAW264.7 cells). These cells were then treated with LPS (24 h, 1 μg/ml) after the preincubation with the miR-106a-5p mimic (n = 5 per group). *B*, the mRNA levels of *Il-6*, *Il-1β*, *Nos2*, *Tnf-α*, and *Ccl2* in LPS (24 h, 1 μg/ml)-treated ST-Pfkfb3-RAW264.7 cells with miR-106a-5p mimic pretreated (n = 6 per group). *C*, IF staining of Casp-1 and Asc in miR-106a-5p mimic-treated ST-Pfkfb3-RAW264.7 cells challenged with vehicle or LPS (1 μg/ml) for 24 h, followed by incubating with ATP (4 mM) for 45 min (n = 3 per group). *D*, the protein levels of full-length Gsdmd and Pro-Il-1β in miR-106a-5p mimic-treated ST-Pfkfb3-RAW264.7 cells challenged with vehicle or LPS (1 μg/ml) for 24 h, followed by incubating with ATP (4 mM) for 45 min (n = 2 per group). The quantification of Western blot images was performed using Image J software (n = 4 biological replicates). Values are presented as means ± SD, ∗∗∗*p* < 0.001 (one-way ANOVA with Tukey’s post hoc test). Asc, apoptosis-associated speck-like protein with a caspase recruitment domain; Ccl2, chemokine ligand 2; ECAR, extracellular acidification rate; Il-1β, interleukin-1β; IL-6, interleukin-6; LPS, lipopolysaccharide; Nos2, nitric oxide synthase 2; PFKFB3, 6-phosphofructo-2-kinase/fructose-2,6-biphosphatase 3; TNF, tumor necrosis factor.

To ascertain that the anti-inflammatory and antipyroptotic effects induced by miR-106a-5p are directly linked to Pfkfb3 inhibition, we introduced miR-106a-5p into RAW264.7 cells overexpressing Pfkfb3 (ST-Pfkfb3-RAW264.7 cells) *via* a stable transfection process using a *Pfkfb3* lentiviral vector (Fig. S6, *A* and *B*). Our data showed that miR-106a-5p was unable to inhibit the cellular glycolytic capacity in ST-Pfkfb3-RAW264.7 cells (Fig. 6*A*). The mRNA levels of inflammatory factors such as *Il-6*, *Il-1β*, *Nos2*, *Tnf-α*, and *Ccl2* were not reduced by the miR-106a-5p mimic in LPS-treated ST-Pfkfb3-RAW264.7 cells (Fig. 6*B*). Furthermore, IF staining and Western blot analysis showed that the increased expression and colocalization of caspase 1 and Asc (Fig. 6*C*), as well as the expression of Gsdmd and Il-1β (Fig. 6*D*) in ST-Pfkfb3-RAW264.7 cells treated with LPS and ATP, could not be alleviated by the overexpression of miR-106a-5p. In summary, these findings demonstrate that Pfkfb3 mitigates the inhibitory effects of miR-106a-5p on the inflammatory response and pyroptosis in LPS-stimulated macrophages.

### Therapeutic effect of miR-106a-5p on LPS-induced sepsis in mice through Pfkfb3 inhibition

**Figure 7:**
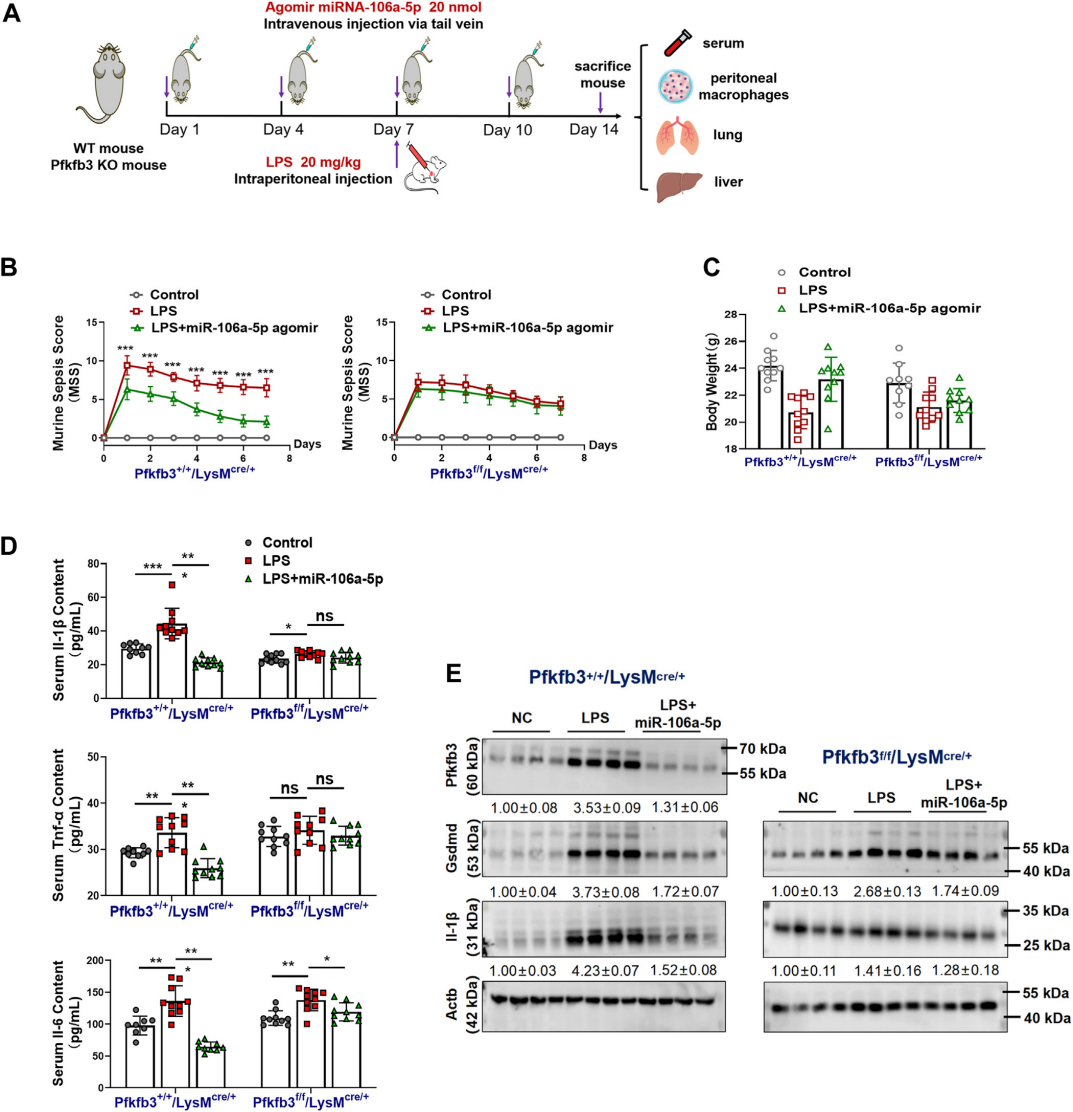
**Therapeutic effect of miR-106a-5p on LPS-induced sepsis in mice *via* Pfkfb3 inhibition.***A*, the schematic diagram of animal study. *B*, the murine sepsis score (MSS) curves of LPS (20 mg/kg)-treated *Pfkfb3*^+/+^/LysM^cre/+^ and *Pfkfb3*^f/f^/LysM^cre/+^ mice with miR-106a-5p agomir (20 nmol per time) pretreated (n = 10 mice per group). *C*, body weight of LPS (20 mg/kg)-treated *Pfkfb3*^+/+^/LysM^cre/+^ and *Pfkfb3*^f/f^/LysM^cre/+^ mice with miR-106a-5p agomir (20 nmol per time) pretreated (n = 10 mice per group). *D*, serum levels of Il-6, Il-1β, and Tnf-α in LPS (20 mg/kg)-treated *Pfkfb3*^+/+^/LysM^cre/+^ and *Pfkfb3*^f/f^/LysM^cre/+^ mice with miR-106a-5p agomir (20 nmol per time) pretreated (n = 10 mice per group). *E*, the protein levels of Pfkfb3, Gsdmd, and Il-1β in mouse peritoneal macrophages from LPS (20 mg/kg)-treated *Pfkfb3*^+/+^/LysM^cre/+^ and *Pfkfb3*^f/f^/LysM^cre/+^ mice with miR-106a-5p agomir (20 nmol per time) pretreated (n = 4 mice per group). Values are presented as means ± SD, ∗*p* < 0.05, ∗∗*p* < 0.01, and ∗∗∗*p* < 0.001 (two-way ANOVA with Bonferroni post hoc tests for B; one-way ANOVA with Tukey’s post hoc test for C and D). Gsdmd, gasdermin D; Il-1β, interleukin-1β; IL-6, interleukin-6; LPS, lipopolysaccharide; PFKFB3, 6-phosphofructo-2-kinase/fructose-2,6-biphosphatase 3; TNF, tumor necrosis factor.

To assess the therapeutic potential of miR-106a-5p *in vivo* and its reliance on *Pfkfb3* inhibition, we established an LPS-induced sepsis model in both *Pfkfb3*^+/+^/LysM^cre/+^ mice (serving as WT controls) and *Pfkfb3*^flox/flox^/LysM^cre/+^ mice. The experimental strategies are outlined in Figure 7*A*. The specific PCR primer sequences and PCR identification of these mice are presented in Table S4 and Fig. S7*A*, respectively. Following intraperitoneal injection of LPS, we continuously monitored the murine sepsis score (MSS) in these mice. Our results showed that treatment with miR-106a-5p agomir significantly reduced the MSS in LPS-stimulated WT control mice, but had a minimal effect on the MSS in LPS-stimulated *Pfkfb3*^flox/flox^/LysM^cre/+^ mice (Fig. 7*B*). Similarly, the miR-106a-5p agomir treatment significantly reversed the LPS-induced decrease in body weight in WT control mice, but not in *Pfkfb3*^flox/flox^/LysM^cre/+^ mice (Fig. 7*C*). Furthermore, the miR-106a-5p agomir therapy reduced the serum levels of Il-1β, Tnf-α, and Il-6 in WT control mice, indicating that miR-106a-5p exerts anti-inflammatory effects *in vivo* (Figs. 7*D* and S7*B*). However, this anti-inflammatory function of miR-106a-5p was lost in *Pfkfb3*^flox/flox^/LysM^cre/+^ mice (Fig. 7*D*). Western blot analysis revealed that the protein level of Pfkfb3 was significantly suppressed in peritoneal macrophages from miR-106a-5p agomir-treated WT mice, providing further evidence for the on-target effects of therapeutic miR-106a-5p *in vivo* (Fig. 7*E*). Additionally, treatment with the miR-106a-5p agomir attenuated the LPS-induced upregulation of Gsdmd and Il-1β levels in macrophages from WT mice. This inhibitory effect of the miR-106a-5p agomir was partially abrogated in macrophages from Pfkfb3^flox/flox^/LysM^cre/+^ mice, suggesting that the suppression of inflammatory macrophage pyroptosis by miR-106a-5p *in vivo* is, at least partially, dependent on Pfkfb3 (Fig. 7*E*).

**Figure 8:**
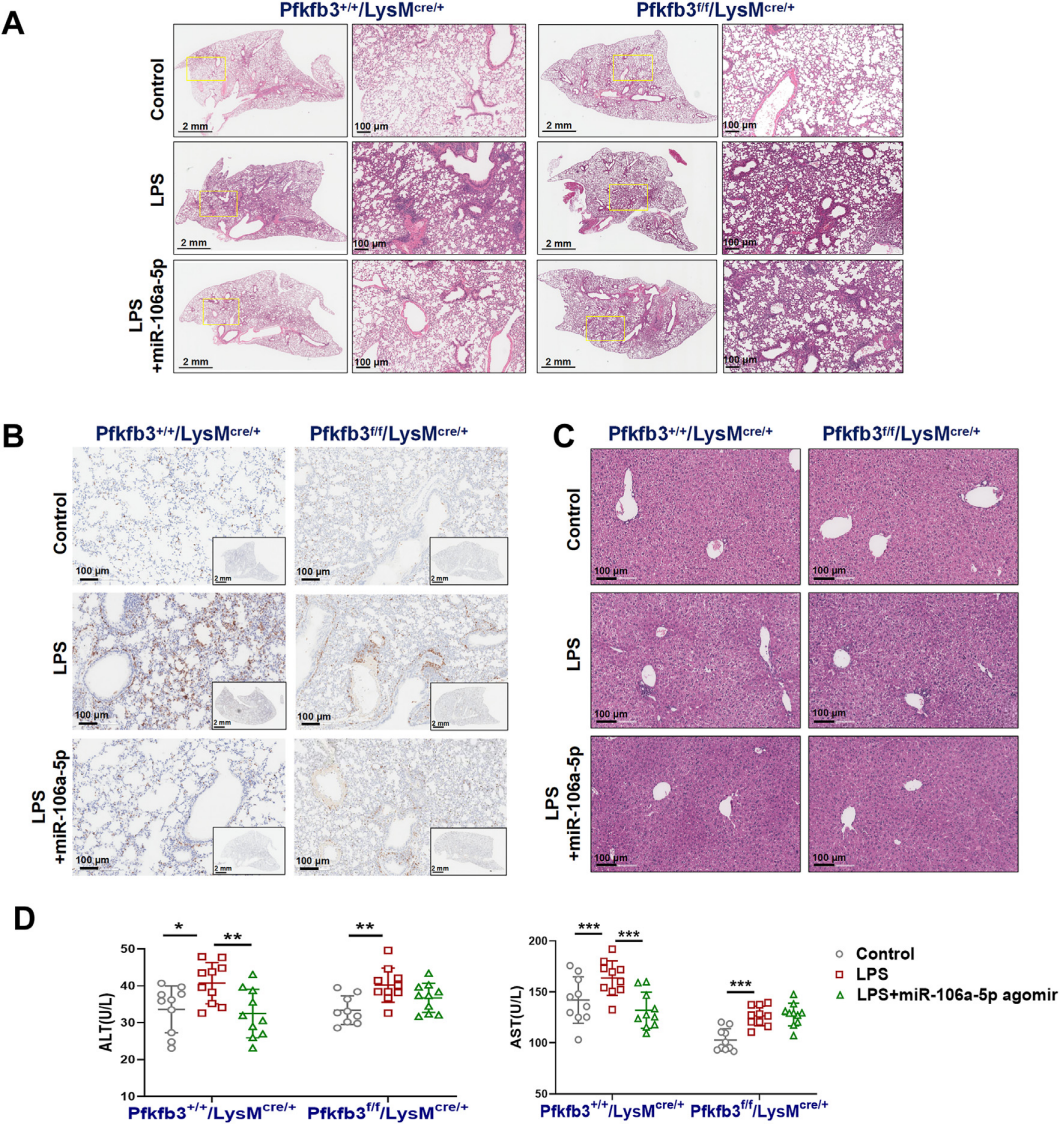
**Therapeutic effects of miR-106a-5p on LPS-induced lung and liver injury in mice *via* Pfkfb3 inhibition.***A*, representative H&E-stained lung sections of LPS (20 mg/kg)-treated *Pfkfb3*^+/+^/LysM^cre/+^ and *Pfkfb3*^f/f^/LysM^cre/+^ mice with miR-106a-5p agomir (20 nmol per time) pretreated (n = 10 mice per group). *B*, immunohistochemistry analyses of F4/80 to indicate the enrichment of macrophage in lungs of LPS (20 mg/kg)-treated *Pfkfb3*^+/+^/LysM^cre/+^ and *Pfkfb3*^f/f^/LysM^cre/+^ mice with miR-106a-5p agomir (20 nmol per time) pretreated (n = 10 mice per group). *C*, representative H&E-stained liver sections of LPS (20 mg/kg)-treated *Pfkfb3*^+/+^/LysM^cre/+^ and *Pfkfb3*^f/f^/LysM^cre/+^ mice with miR-106a-5p agomir (20 nmol per time) pretreated (n = 10 mice per group). *D*, serum levels of alanine transaminase (ALT) and aspartate transaminase (AST) in LPS (20 mg/kg)-treated *Pfkfb3*^+/+^/LysM^cre/+^ and *Pfkfb3*^f/f^/LysM^cre/+^ mice with miR-106a-5p agomir (20 nmol per time) pretreated (n = 10 mice per group). Values are presented as means ± SD, ∗*p* < 0.005 (one-way ANOVA with Tukey’s post hoc test). LPS, lipopolysaccharide; PFKFB3, 6-phosphofructo-2-kinase/fructose-2,6-biphosphatase 3.

To assess the extent of organ injury associated with sepsis, we conducted H&E and F4/80 immunohistochemical staining on lung tissue samples from mice. Our observations revealed that control mice treated with LPS exhibited a significant increase in macrophage content and severe structural alterations in the alveoli, including pulmonary interstitial edema and thickening of the alveolar walls. Remarkably, these defects were significantly ameliorated following treatment with the miR-106a-5p agomir (Fig. 8, *A* and *B*). However, the protective effects of the miR-106a-5p agomir on sepsis-associated lung injury were minimal in *Pfkfb3*^flox/flox^/LysM^cre/+^ mice (Fig. 8, *A* and *B*). In terms of sepsis-associated liver injury, although H&E staining did not reveal significant liver tissue damage (Fig. 8*C*), there was an elevation in the serum levels of liver function-related markers, including alanine aminotransferase and aspartate aminotransferase, in LPS-stimulated WT mice (Fig. 8*D*). This suggested the presence of liver dysfunction. The administration of the miR-106a-5p agomir significantly attenuated this LPS-induced liver dysfunction in WT mice, but not in Pfkfb3 KO mice (Fig. 8*D*). Collectively, these findings underscore the protective effect and therapeutic potential of miR-106a-5p in an LPS-induced sepsis mouse model. This protective effect is closely associated with its regulation of Pfkfb3.

## Discussion

In the present study, we observed the upregulation of Pfkfb3 and downregulation of miR-106a-5p in blood monocytes from patients with early sepsis and from an LPS-induced sepsis mouse model for the first time. We also demonstrated the anti-inflammatory and anti-pyroptotic effect of miR-106a-5p in LPS-induced sepsis in mice. Treatment of mice with miR-106a-5p agomir reduces the severity of LPS-induced sepsis and the associated lung and liver injuries by decreasing the glycolytic level, inflammatory response, and pyroptosis in macrophages. The improved condition following miR-106a-5p treatment is attributed to the reduced expression of Pfkfb3.

In the acute phase of sepsis, a strong immune response aims to eliminate the pathogen (1, 2, 6). However, excessive inflammatory response can trigger a cytokine storm, exacerbating sepsis and causing organ damage (1, 3, 5). Monocytes and macrophages, early responders to septic infection, regulate inflammatory responses (7, 25). Activated macrophages, characterized by enhanced glycolytic flux, rapidly generate ATP and biosynthetic intermediates to support cytokine production (9, 25, 26). PFKFB3, a key glycolytic enzyme, has been shown to promote the overactivation of macrophages and the development of sepsis (13, 14, 15). Inhibition of PFKFB3 can improve sepsis induced by LPS or cecal ligation, suggesting its potential as a treatment target (17, 27). In our investigation, we discovered that the expression of PFKFB3 is significantly elevated in the blood mononuclear cells of patients with sepsis. This observation corroborates previous findings from animal studies and highlights the potential of PFKFB3 as a sepsis biomarker. Several studies have demonstrated that the transcription factors hypoxia-inducible factor 1α and zinc fingers and homeoboxes 2 can transcriptionally upregulate Pfkfb3 in the context of sepsis (17, 28). In addition to these transcriptional mechanisms, our research found that in sepsis, Pfkfb3 upregulation in monocytes and macrophages is also posttranscriptionally regulated by miR-106a-5p, with a decrease in miR-106a-5p during sepsis leading to the stabilization of *Pfkfb3* mRNA.

In this study, we performed a comprehensive screening for differentially expressed genes and miRNAs in sepsis using publicly available microarray data from the GEO database. Our analysis revealed that miR-106a-5p was the most significantly downregulated miRNA in sepsis. To further validate our findings, we collected blood monocytes from sepsis patients during the inflammatory immune sensitivity stage and demonstrated a downregulation of miR-106a-5p expression. Previous research has shown that the CircTLK1–miR-106a-5p/HMGB1 axis plays an anti-inflammatory role in LPS-stimulated HK-2 cells and is effective in treating a cecum ligation and puncture) sepsis mouse model (29). Our current study suggests that miR-106a-5p can inhibit macrophage inflammatory response by suppressing glycolysis, further elucidating its antisepsis mechanism. However, the underlying mechanism of miR-106a-5p downregulation in monocytes/macrophages during sepsis warrants further clarification.

Our detailed analysis has confirmed that *Pfkfb3* mRNA is a legitimate target gene of miR-106a-5p, as evidenced by the study of protein expression and *Pfkfb3* 3′-UTR activity. Functional experiments have demonstrated that miR-106a-5p can inhibit LPS-induced production of ROS, cell pyroptosis, and the inflammatory response in macrophages through Pfkfb3 inhibition. As for the mechanism by which PFKFB3 controls ROS production, previous studies have indicated that the downregulation of PFKFB3 can facilitate an increased glucose flux into the pentose phosphate pathway. This, in turn, boosts the generation of the ROS scavenger NADPH, ultimately leading to a reduction in ROS production (14, 16). This mechanism may also be operative in LPS-treated macrophages. Our *in vivo* study has shown that the knockout of *Pfkfb3* in macrophages only partially mitigates the antisepsis effects of miR-106a-5p agomir. This suggests that other mechanisms, in addition to the inhibition of *Pfkfb3*, may be involved in the antisepsis effects of miR-106a-5p. These mechanisms will be the subject of future investigations.

Pyroptosis, a proinflammatory cell death caused by caspase-1 during infection, is increasingly seen as a key mechanism of sepsis, mediated by NLRP3 inflammasome activation (30, 31). The NLRP3 inflammasome, a multiprotein complex, responds to infectious and noninfectious stimuli, activating caspase-1, and caspase-4/5/11, which in turn activate Gsdmd (4, 32, 33, 34). This leads to the formation of pores in the cell membrane, causing cell death and the release of inflammatory mediators like IL-1β and IL-18 (8, 30, 32). Therefore, activation of the CASP-1–GSDMD pathway or upregulation of inflammasome components like NLRP3, ASC, and IL-1β are considered classic signs of pyroptosis. Based on the highly inflammation-dependent mode of pyroptosis and the effective role of miR-106-5p in inhibiting macrophage inflammatory responses, we began to explore the impact of the miR-106a-5p–PFKFB3 axis on macrophage pyroptosis. We found that LPS-stimulated macrophages are more prone to pyroptosis than traditional apoptosis. Transfection with miR-106a-5p mimic or si*Pfkfb3* significantly reduced pyroptosis markers in inflammatory macrophages. Recent studies have demonstrated that the activation of the NLRP3 inflammasome and the induction of pyroptosis are dependent on the level of glycolysis in many cell types (33, 35, 36). In sepsis, particularly during the acute phase, a positive feedback loop may exist between pyroptosis and inflammation. Excessive inflammatory response can stimulate macrophage pyroptosis, leading to the release of large quantities of intracellular inflammatory factors and exacerbating inflammation. Our research suggests that miR-106a-5p treatment can disrupt this positive feedback loop by targeting PFKFB3-mediated glycolysis. Although the protective role of miR-106a-5p observed in LPS-induced cell and animal models may suggest a partial inhibition of macrophage pyroptosis, much work remains to identify the potential secondary mechanisms that allow miR-106a-5p to confer protection against sepsis.

Compared to traditional small-molecule therapeutics, miRNA-based pharmaceuticals offer several distinct advantages, including the potential for rapid and cost-effective development, high specificity for their intended targets, and minimal off-target effects on other physiological functions (18, 37, 38). Agomirs, chemically modified miRNA mimics, are widely utilized in *in vivo* miRNA experiments and have demonstrated considerable therapeutic potential in various disease models (39, 40). In this study, we investigated the therapeutic potential of miR-106a-5p agomir in a mouse model of LPS-induced sepsis. Our results demonstrated that treatment with miR-106a-5p agomir effectively suppressed sepsis by downregulating serum levels of inflammatory factors, mitigating lung inflammatory injury, and inhibiting the enrichment of lung macrophages. Furthermore, the miR-106a-5p agomir treatment significantly decreased the expression levels of Gsdmd and Il-1β in the peritoneal macrophages of mice, leading to a reduction in inflammatory macrophage pyroptosis. However, it is important to consider the existence of a therapeutic window when utilizing miR-106a-5p-based therapy for the treatment of sepsis. Administration during the acute phase may alleviate excessive inflammatory responses, while administration at later stages may exacerbate immune suppression. Further studies are necessary to definitively elucidate the potential side effects of miR-106a-5p–based therapy *in vivo*.

In summary, our study demonstrated a downregulation of miR-106a-5p expression and an upregulation of PFKFB3 expression in septic patients, which was further validated using an *in vitro* inflammatory macrophage model. We also discovered that miR-106a-5p negatively regulates Pfkfb3 expression, and overexpression of miR-106a-5p inhibits glucose metabolism, pyroptosis, and inflammatory response in macrophages through a Pfkfb3 inhibition-dependent mechanism. Finally, we demonstrated the therapeutic efficacy of miR-106a-5p in a mouse model of LPS-induced sepsis, suggesting that targeting miR-106a-5p in inflammatory macrophages may have potential therapeutic applications for the treatment of sepsis. Collectively, our data provide proof of concept for the development of a miRNA-based therapy against sepsis.

## Experimental procedures

### Meta-analysis and human studies

In our quest to identify new targets and miRNA markers for sepsis, a meta-analysis encompassing various types of studies was performed. GEO series (GSE) related to sepsis were retrieved from GEO database. Following a meticulous screening process, we selected series that met specific criteria: each series had to comprise at least three sepsis samples and three healthy control samples, and the generation of mRNA data had to involve the use of the Affymetrix HG-U133 platform or a higher version. A summary of the selected microarray GEO series data is presented in Table S1. The workflow of our meta-analysis, which has been previously described (41), is shown in Fig. S1. The collection of human samples was approved by the Clinical Research Ethics Committee of the Second Affiliated Hospital of Guangzhou Medical University. Human studies abide by the Declaration of Helsinki principles.

### Cell isolation and culture

The mouse monocytic cell line, RAW264.7 (ATCC Cat# TIB-71), was obtained from American Type Culture Collection. These cells were cultured in Dulbecco’s modified Eagle’s medium, supplemented with 10% fetal bovine serum (Gibco) and 1% streptomycin sulfate and penicillin sodium (Gibco). The culture was maintained at 37 °C in a humidified atmosphere containing 5% CO_2_ and 95% air. ZsGreen-Puro–labeled *Pfkfb3* lentiviral and control vectors were established by Shanghai HanBio Company. The RAW264.7 cells were transduced with the *Pfkfb3* lentiviral construct, and recombinant cells were selected using puromycin, resulting in the creation of the ST-*Pfkfb3*-RAW264.7 cell line. Murine BMDMs were isolated from the tibia and femur of 6-week-old male C57BL/6 mice. These cells were cultured in RPMI-1640 medium, supplemented with 10% fetal bovine serum, 1% penicillin-streptomycin, 0.1% β-mercaptoethanol, and 20% L929 cell culture supernatant. Peripheral blood mononuclear cells were isolated from the blood of both patients with sepsis and healthy controls using the separation kit P8680 (Solarbio). Lastly, peritoneal macrophages were isolated from the peritoneal lavage fluid of both WT and *Pfkfb3* knockdown mice by peritoneal lavage.

### Cell treatment and transfection

RAW264.7 cells in the logarithmic growth phase or BMDMs that had been cultured for 7 days were seeded into 6-, 12-, or 96-well plates. Subsequently, the cells were transfected with either a miR-106a-5p mimic or *Pfkfb3* siRNA (RiboBio) for a duration of 72 h using Lipofectamine RNAiMAX reagent (Invitrogen) and Opti-minimal essential medium (Gibco). Forty-eight hours after the transfection, the cells were stimulated with 1 μg/ml LPS (Sigma) for an additional 24 h. For experiments related to the activation of the NLRP3 inflammasome, the cells were exposed to 4 mM ATP for an additional 45 min following the LPS treatment.

### RNA isolation and quantitative real-time PCR analysis

Total RNA and miRNA were extracted using the TRIzol reagent (Invitrogen) and the miRcute miRNA isolation kit (Vazyme), respectively. The extracted RNA was quantified using a NanoDrop microvolume spectrophotometer (Thermo Fisher Scientific). The synthesis of single-stranded complementary DNA was performed using the PrimeScript RT reagent kit (TaKaRa) and the miRNA first strand complementary DNA synthesis kit (Vazyme), respectively. Real-time quantitative PCR analyses were performed on a CFX96 touch real-time pcr detection system (Bio-Rad) using SYBR Green Pro Taq HS (Accurate). The relative expression levels of the target genes were calculated using the ^ΔΔ^Ct method. The primer sequences are provided in Tables S2 and S3.

### Protein isolation and Western blot analysis

Total protein was extracted using radio-immunoprecipitation lysis buffer (Invitrogen), which was supplemented with 1% 100 mM PMSF (Thermo Fisher Scientific). The protein concentration was then quantified using the bicinchoninic acid protein assay kit (Thermo Fisher Scientific). The protein expression levels were then analyzed using a Western blot assay. Equal quantities of protein (10 μg) were separated using SDS-PAGE on a 10% gel and then transferred onto a polyvinylidene fluoride membrane. The membrane was blocked and incubated overnight at 4 °C with primary antibodies against Pfkfb3 (1:5000, Abcam Cat# ab181861), Actb (1:1000, Santa Cruz Cat# sc-8432), Gsdmd (1:1000, Abcam Cat# ab209845), and Il-1β (1:1000, Cell Signaling Technology Cat# 12242S). This was followed by an incubation with secondary anti-mouse or anti-rabbit antibodies (1:5000, Cell Signaling Technology, Cat# 7076, or Cat# 7074) at room temperature. Blots were developed using electro-chemiluminescence detection substrates (Engreen Biosystem) and images were captured using a ChemiDoc MP Imaging System (Bio-Rad). In most cases, membranes were either cut horizontally or stripped using the Western Blot stripping buffer (Thermo Fisher Scientific) and reprobed. In some special cases, the same batch of samples was reloaded onto another gel for the detection of loading controls. The quantification of Western blot images was performed using Image J software (https://imagej.net/ij/download.html). Briefly, the intensity of each band was measured and normalized by subtracting the background value and dividing by the loading control value. The fold change was then calculated by dividing the normalized intensity by the mean value of the control group.

### MiRNA response element site identification and luciferase assay

The miRNA response element of miR-106a-5p within the 3′-UTR of *PFKFB3* mRNA was identified using the TargetScan database. Reporter plasmids were constructed by cloning the WT or mut 3′UTR segment downstream of the firefly luciferase gene within the Pezx-MT06 vector (Genecopoeia). HEK-293T, BMDMs, and RAW264.7 cells were cultured in white 96-well plates and cotransfected with varying concentrations of miR-106a-5p mimic and either WT-*Pfkfb3* or mut-*Pfkfb3* reporter plasmids. The luciferase activity was measured using a commercial dual-luciferase reporter assay kit (Promega) and a Spark microplate reader (Tecan), following the manufacturer’s protocols.

### Metabolic measurements

ECARs were measured using a Seahorse XF96e Extracellular Flux analyzer (Agilent). Briefly, cells transfected with either miR-106a-5p mimic or *Pfkfb3* siRNA were seeded into an XF96 cell culture plate at a density of 5000 cells per well and incubated overnight with LPS. Following 24 h of LPS treatment, the medium was replaced with Seahorse assay medium and the plates were incubated for 1 h in a non-CO2 incubator at 37 °C. Three basal measurements were taken, followed by three consecutive measurements executed by the sequential addition of glucose (10 mM), the ATP synthase inhibitor oligomycin (1.5 μM), and the glycolysis inhibitor 2-deoxy-D-glucose 100 mM) to determine basal and maximum ECAR. The basal ECAR was calculated as the average ECAR value during the basal measurement cycle, prior to the addition of any compounds. Glycolysis was calculated as the difference between the ECAR after adding glucose and the nonglycolytic acidification, which is the ECAR after adding 2-deoxy-D-glucose. Glycolytic capacity was calculated as the difference between the ECAR after adding oligomycin, which stimulates glycolysis, and the basal ECAR reading. Glycolytic reserve was calculated as the difference between the ECAR after adding oligomycin and the ECAR after adding glucose. Finally, ECAR values were normalized to the cell number.

### Glucose uptake assay

For the glucose uptake assay, cells were initially washed twice with glucose-free Krebs-Ringer bicarbonate buffer (pH 7.4) and then preincubated with the same buffer for 15 min at 37 °C. Subsequently, the buffer was replaced with Krebs-Ringer bicarbonate buffer that was supplemented with 600 mM 2-Deoxy-2- [(7-nitro-2,1,3-benzoxadiazol-4-yl)amino]-D-glucose (Thermo Fisher Scientific), and the cells were incubated at 37 °C for an additional 30 min. Following three washes with PBS, the fluorescence intensity of the samples was immediately measured using a microplate reader (Tecan) at a wavelength of 465/540 nm. Finally, the protein concentration in each cell sample was determined using the bicinchoninic acid assay, which was used to normalize the glucose uptake capacity.

### Assay of intracellular ROS content

Intracellular ROS levels were determined using either a DCFH-DA fluorescent probe (Beyotime) or DHE (solarbio). Specifically, intracellular ROS can oxidize the nonfluorescent DCFH to generate fluorescent dichlorofluorescein. Additionally, ROS can oxidize DHE to ethidium oxide, which can be incorporated into chromosomal DNA to produce red fluorescence. Cells were pretreated with either DCFH-DA or DHE working solution and then incubated at 37 °C with 5% CO_2_ for 30 min. Fluorescence signals were subsequently detected using a FACScan^TM^ flow cytometer system (BD Biosciences) at a wavelength of 488 nm. Finally, data were processed using FlowJo Version 10.8.1 (https://www.flowjo.com/).

### ATP content detection

Total ATP production was assessed by measuring the fluorescence intensity with an ATP assay kit (Beyotime). Briefly, the ATP detection reagent was diluted in a 1:9 ratio using the ATP dilution buffer. Subsequently, 100 μl of the detection solution was added to a white 96-well plate and incubated at room temperature for 3 to 5 min to allow for the consumption of background ATP. Following this, the solution was incubated with either the samples or standards. The fluorescence intensity was then immediately measured using a luminometer reader (Tecan).

### Immunofluorescent staining

For immunofluorescent staining, cells were plated onto cell slides within a 12-well plate. The expression levels and colocalization of pyroptosis-associated proteins were determined by first fixing the cells with 4% paraformaldehyde. Following permeabilization with 0.1% Triton X-100 and blocking with 10% goat serum, the cells were incubated overnight at 4 °C with primary antibodies against caspase-1 (1:200, Proteintech Cat# 22915-1-AP) and Asc (1:200, Santa Cruz Cat# sc-514414). Corresponding secondary antibodies conjugated to Alexa Fluor 488 (anti-mouse), Alexa Fluor 555 (anti-rat), or Alexa Fluor 594 (anti-rabbit) were then applied (Thermo Fisher Scientific). The nuclei were stained with 4',6-diamidino-2-phenylindole (Cell Signaling Technology). Finally, all images were captured using a confocal fluorescence microscopy system (Leica).

### ELISA assay

The levels of Il-6, Il-1β, and Tnf-α in either the cell culture supernatant or mouse serum were analyzed using ELISA kits (Proteintech Cat# KE10007, KE10003, and KE10002, respectively), in accordance with the manufacturer’s instructions. Briefly, 100 μl of the test samples were added to each well and incubated at 37 °C for a duration of 2 h. Following this, the samples were washed and then sequentially incubated with antibodies and horseradish peroxidase-labeled streptomycin for an additional hour. Immunoreactive signals were developed using a 3,3′,5,5′-Tetramethylbenzidine solution to induce a color reaction. The absorbance was immediately measured at 450/630 nm using a microplate reader (Tecan).

### Flow cytometric analysis of pyroptosis and apoptosis

The status of live cells was evaluated using a caspase-3 activity and apoptosis detection kit (Beyotime). Specifically, the culture medium was collected before cells were digested with trypsin. The cell pellet was then mixed with the medium and resuspended. Following two washes with PBS, the cells were incubated with 194 μl of binding buffer, 5 μl of annexin V-mCherry, and 1μl of GreenNuc caspase-3 substrate. This incubation was carried out at room temperature in the dark for a duration of 30 min. Subsequently, the cells were analyzed using a FAC-scan flow cytometer (BD Biosciences) on the FITC/phycoerythrin channels. Finally, the data were processed using FlowJo version 10.8.1.

### Transmission electron microscopy

Cell pyroptosis was examined using transmission electron microscopy, with the assistance of the Sinoma Electron Microscope Center. The cells were harvested and fixed in ice-cold 2.5% glutaraldehyde for a duration of 2 h. Following this, the samples were postfixed in 1% OsO4 for an additional hour, dehydrated through a series of ethanol washes, and then embedded in epoxy resin. Ultrathin sections, measuring 60 nm, were prepared and double-stained with uranyl acetate and lead citrate. These sections were subsequently examined using a transmission electron microscope.

### Animal study

The animal studies conducted were approved by the Institutional Animal Care and Use Committee at Guangzhou Medical University. Floxed *Pfkfb3* (*Pfkfb3*^flox/flox^) mice were generously provided by Dr Yuqing Huo from Augusta University. A homozygous deficiency of *Pfkfb3*, specific to myeloid cells (*Pfkfb3*^flox/flox^/LysM^cre/+^, hereafter referred to as *Pfkfb3*^f/f^/LysM^cre/+^), was achieved by cross-breeding LysM^cre/+^ transgenic mice (Stock Number: 004718, The Jackson Laboratory) with *Pfkfb3*^flox/flox^ mice. All mice were on a C57BL/6 background.

To minimize estrogen-dependent fluctuations in response to sepsis, only male mice were used for evaluating sepsis. Male *Pfkfb3*^f/f^/LysM^cre/+^ and *Pfkfb3*^+/+^/LysM^cre/+^ mice, aged between 6 and 7 weeks, were randomized into three groups, with ten mice per group: control, LPS-treated, and miR-106a-5p–treated. Mice in the control and LPS-treated groups were intravenously administered with PBS, while those in the miR-106a-5p–treated group received intravenous administration of miR-106a-5p agomir (20 nmol/administration) twice a week *via* the tail vein. One week after agomir treatment, mice in the LPS-treated and miR-106a-5p–treated groups were intraperitoneally injected with LPS (20 mg/kg body weight). The animals were treated with LPS once every 3 days for a duration of 2 weeks and were euthanized on the third day following the last dose administration. The state and behavior of the mice were evaluated daily during this period using the MSS. Whole blood, lung, and liver samples were collected for serological and histopathological analyses, respectively. Mouse peritoneal macrophages were also processed for Western blot analysis.

### Statistical analysis

All data are represented as the mean ± SD. Comparisons between two groups were carried out using a two-sided unpaired Student’s *t* test. Multiple comparisons among three or more groups were performed using one-way ANOVA, followed by Tukey’s *post hoc* test or two-way ANOVA, followed by Bonferroni’s post hoc test. All data were analyzed and visualized using GraphPad Prism 8.0 software (https://www.graphpad.com/features). A *p* value of less than 0.05 was considered statistically significant (∗*p* < 0.05, ∗∗*p* < 0.01, and ∗∗∗*p* < 0.001). Each experiment was independently repeated at least three times. The number of biological replicates in each group is shown in the Figure legends.

## Data availability

All data that support the findings in this study are available from the corresponding author upon reasonable request.

## Supporting information

This article contains supporting information.

## Conflict of interest

The authors declare that they have no conflict of interests with the contents of this article.

## References

[1] Gotts J.E., Matthay M.A. (2016). Sepsis: pathophysiology and clinical management. BMJ.

[2] Salomão R., Ferreira B.L., Salomão M.C., Santos S.S., Azevedo L.C.P., Brunialti M.K.C. (2019). Sepsis: evolving concepts and challenges. Braz. J. Med. Biol. Res..

[3] Cheng Z., Abrams S.T., Toh J., Wang S.S., Wang Z., Yu Q. (2020). The critical roles and mechanisms of immune cell death in sepsis. Front. Immunol..

[4] Wen X., Xie B., Yuan S., Zhang J. (2022). The “self-sacrifice” of ImmuneCells in sepsis. Front. Immunol..

[5] Lelubre C., Vincent J.L. (2018). Mechanisms and treatment of organ failure in sepsis. Nat. Rev. Nephrol..

[6] Chen X., Liu Y., Gao Y., Shou S., Chai Y. (2021). The roles of macrophage polarization in the host immune response to sepsis. Int. Immunopharmacol.

[7] Kumar V. (2018). Targeting macrophage immunometabolism: dawn in the darkness of sepsis. Int. Immunopharmacol..

[8] Karakike E., Giamarellos-Bourboulis E.J. (2019). Macrophage activation-like syndrome: a distinct entity leading to early death in sepsis. Front. Immunol..

[9] Viola A., Munari F., Sánchez-Rodríguez R., Scolaro T., Castegna A. (2019). The metabolic signature of macrophage responses. Front. Immunol..

[10] Thapa B., Lee K. (2019). Metabolic influence on macrophage polarization and pathogenesis. BMB Rep..

[11] Van den Bossche J., O'Neill L.A., Menon D. (2017). Macrophage immunometabolism: where are we (going)?. Trends Immunol..

[12] De Jesus A., Keyhani-Nejad F., Pusec C.M., Goodman L., Geier J.A., Stoolman J.S. (2022). Hexokinase 1 cellular localization regulates the metabolic fate of glucose. Mol. Cell.

[13] Guo S., Li A., Fu X., Li Z., Cao K., Song M. (2022). Gene-dosage effect of Pfkfb3 on monocyte/macrophage biology in atherosclerosis. Br. J. Pharmacol..

[14] Xiao M., Liu D., Xu Y., Mao W., Li W. (2023). Role of PFKFB3-driven glycolysis in sepsis. Ann. Med..

[15] Jiang H., Shi H., Sun M., Wang Y., Meng Q., Guo P. (2016). PFKFB3-driven macrophage glycolytic metabolism is a crucial component of innate antiviral defense. J. Immunol..

[16] Shi L., Pan H., Liu Z., Xie J., Han W. (2017). Roles of PFKFB3 in cancer. Signal Transduct. Target. Ther..

[17] Wang Z., Kong L., Tan S., Zhang Y., Song X., Wang T. (2020). Zhx2 accelerates sepsis by promoting macrophage glycolysis via Pfkfb3. J. Immunol..

[18] Ambros V. (2004). The functions of animal microRNAs. Nature.

[19] Lagos-Quintana M., Rauhut R., Yalcin A., Meyer J., Lendeckel W., Tuschl T. (2002). Identification of tissue-specific microRNAs from mouse. Curr. Biol..

[20] Benz F., Roy S., Trautwein C., Roderburg C., Luedde T. (2016). Circulating MicroRNAs as biomarkers for sepsis. Int. J. Mol. Sci..

[21] Du W., Fan L., Du J. (2023). Neuroinflammation-associated miR-106a-5p serves as a biomarker for the diagnosis and prognosis of acute cerebral infarction. BMC Neurol..

[22] Hu J., Xie C., Xu S., Pu Q., Liu H., Yang L. (2023). Liver fibrosis-derived exosomal miR-106a-5p facilitates the malignancy by targeting SAMD12 and CADM2 in hepatocellular carcinoma. PLoS One.

[23] He Q.Y., Wang G.C., Zhang H., Tong D.K., Ding C., Liu K. (2016). miR-106a-5p suppresses the proliferation, migration, and invasion of osteosarcoma cells by targeting HMGA2. DNA Cell Biol..

[24] Simon-Molas H., Arnedo-Pac C., Fontova P., Vidal-Alabró A., Castaño E., Rodríguez-García A. (2018). PI3K-Akt signaling controls PFKFB3 expression during human T-lymphocyte activation. Mol. Cell. Biochem..

[25] Hsieh J.Y., Smith T.D., Meli V.S., Tran T.N., Botvinick E.L., Liu W.F. (2017). Differential regulation of macrophage inflammatory activation by fibrin and fibrinogen. Acta Biomater..

[26] Corcoran S.E., O'Neill L.A. (2016). HIF1α and metabolic reprogramming in inflammation. J. Clin. Invest..

[27] Ruiz-García A., Monsalve E., Novellasdemunt L., Navarro-Sabaté A., Manzano A., Rivero S. (2011). Cooperation of adenosine with macrophage Toll-4 receptor agonists leads to increased glycolytic flux through the enhanced expression of PFKFB3 gene. J. Biol. Chem..

[28] Min J., Zeng T., Roux M., Lazar D., Chen L., Tudzarova S. (2021). The role of HIF1α-PFKFB3 pathway in diabetic retinopathy. J. Clin. Endocrinol. Metab..

[29] Xu H.P., Ma X.Y., Yang C. (2021). Circular RNA TLK1 promotes sepsis-associated acute kidney injury by regulating inflammation and oxidative stress through miR-106a-5p/HMGB1 axis. Front. Mol. Biosci..

[30] Demarco B., Danielli S., Fischer F.A., Bezbradica J.S. (2022). How pyroptosis contributes to inflammation and fibroblast-macrophage cross-talk in rheumatoid arthritis. Cells.

[31] Yu P., Zhang X., Liu N., Tang L., Peng C., Chen X. (2021). Pyroptosis: mechanisms and diseases. Signal Transduct Target Ther..

[32] Burdette B.E., Esparza A.N., Zhu H., Wang S. (2021). Gasdermin D in pyroptosis. Acta Pharm. Sin. B.

[33] He Y., Hara H., Núñez G. (2016). Mechanism and regulation of NLRP3 inflammasome activation. Trends Biochem. Sci..

[34] Robinson N., Ganesan R., Hegedűs C., Kovács K., Kufer T.A., Virág L. (2019). Programmed necrotic cell death of macrophages: focus on pyroptosis, necroptosis, and parthanatos. Redox Biol..

[35] Kayagaki N., Stowe I.B., Lee B.L., O'Rourke K., Anderson K., Warming S. (2015). Caspase-11 cleaves gasdermin D for non-canonical inflammasome signalling. Nature.

[36] Renaudin F., Orliaguet L., Castelli F., Fenaille F., Prignon A., Alzaid F. (2020). Gout and pseudo-gout-related crystals promote GLUT1-mediated glycolysis that governs NLRP3 and interleukin-1β activation on macrophages. Ann. Rheum. Dis..

[37] Barile L., Vassalli G. (2017). Exosomes: therapy delivery tools and biomarkers of diseases. Pharmacol. Ther..

[38] Traber G.M., Yu A.M. (2023). RNAi-based therapeutics and novel RNA bioengineering technologies. J. Pharmacol. Exp. Ther..

[39] He X.C., Wang J., Du H.Z., Liu C.M., Teng Z.Q. (2022). Intranasal administration of Agomir-let-7i improves cognitive function in mice with traumatic brain injury. Cells.

[40] Cai Z., Li J., Zhuang Q., Zhang X., Yuan A., Shen L. (2018). MiR-125a-5p ameliorates monocrotaline-induced pulmonary arterial hypertension by targeting the TGF-β1 and IL-6/STAT3 signaling pathways. Exp. Mol. Med..

[41] Chen Y., Zhou Y., Han F., Zhao Y., Tu M., Wang Y. (2020). A novel miR-1291-ERRα-CPT1C axis modulates tumor cell proliferation, metabolism and tumorigenesis. Theranostics.

