# Supplementary material for: MiR-106a-5p targets PFKFB3 and improves sepsis through regulating macrophage pyroptosis and inflammatory response
Source: J Biol Chem. 2024 May 3;300(6):107334. doi: 10.1016/j.jbc.2024.107334 (PMC11190718; doi:10.1016/j.jbc.2024.107334)
Supplement: Supporting information [file mmc1.docx]

**MiR-106a-5p targets PFKFB3 and improves sepsis through regulating macrophage pyroptosis and inflammatory response**

**Supporting information**

**Figures S1 to S6**

Fig S1. The workflow of meta-analysis.

Fig S2. The summary of down-regulated miRNAs and differential expression of PFKFB3 in a series of GSE data.

Fig S3. The mRNA levels of glycolytic enzymes and ROS levels in LPS-treated macrophages with miR-106a-5p mimic or si*Pfkfb3* pre-transfected.

Fig S4. Flow cytometry analysis of apoptosis and pyroptosis of LPS and ATP-treated macrophages with miR-106a-5p pretreated.

Fig S5. The quantification of Western blot images in Fig 5A-B and their replicate samples.

Fig S6. Verification of the efficiency of stably high expressed PFKFB3 in RAW264.7 cell line.

Fig S7. Genotyping of Pfkfb3^flox/flox^/LysM^cre/+^ mice and standard curve of ELISA analysis.

**Tables S1 to S4**

Table S1. miRNAs that potentially target PFKFB3

Table S2. The summarization of selected Microarrays in GSE database.

Table S3. Sequences of primers for quantitative RT-PCR.

Table S4. Sequences of primers for miRNA quantitative RT-PCR analysis.

Table S5. Sequences of primers for PCR identification of Pfkfb3^+/+^/LysM^cre/+^ and Pfkfb3^flox/flox^/LysM^cre/+^ mice.

**
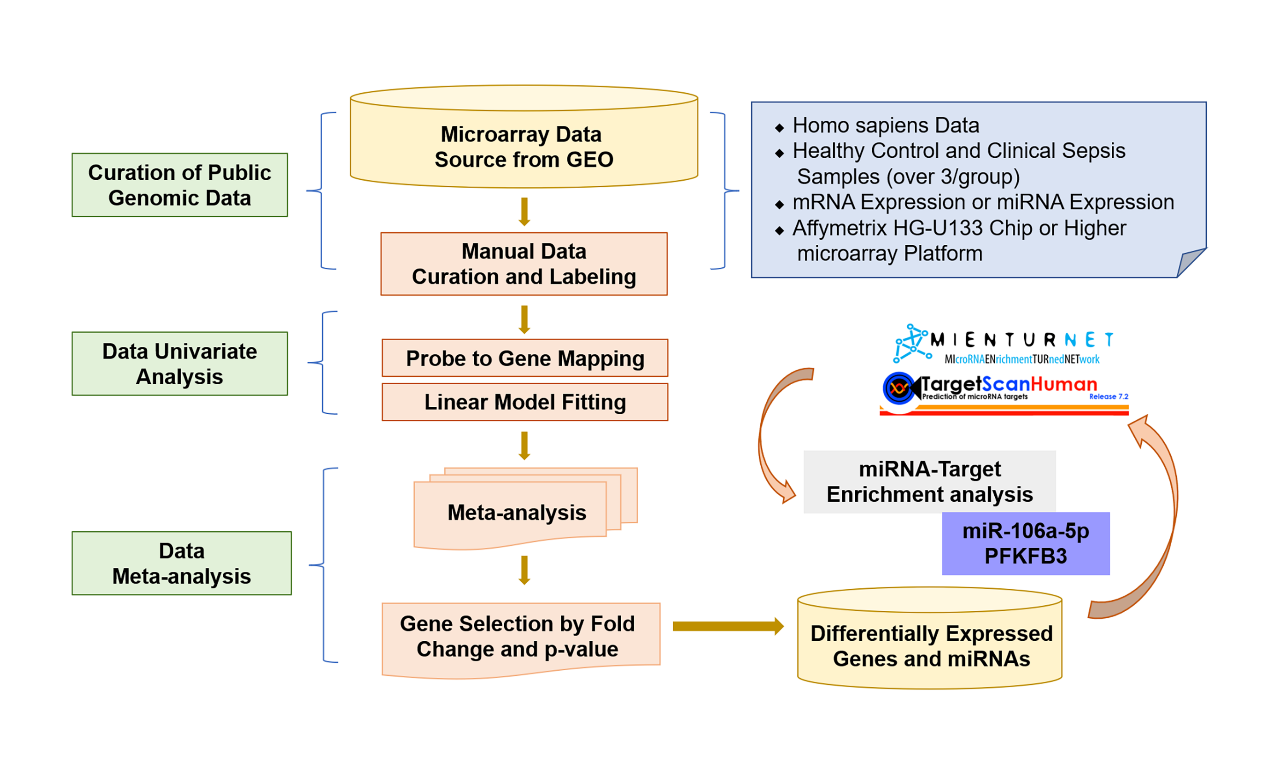
**

**Figure S1.** The workflow of meta-analysis.


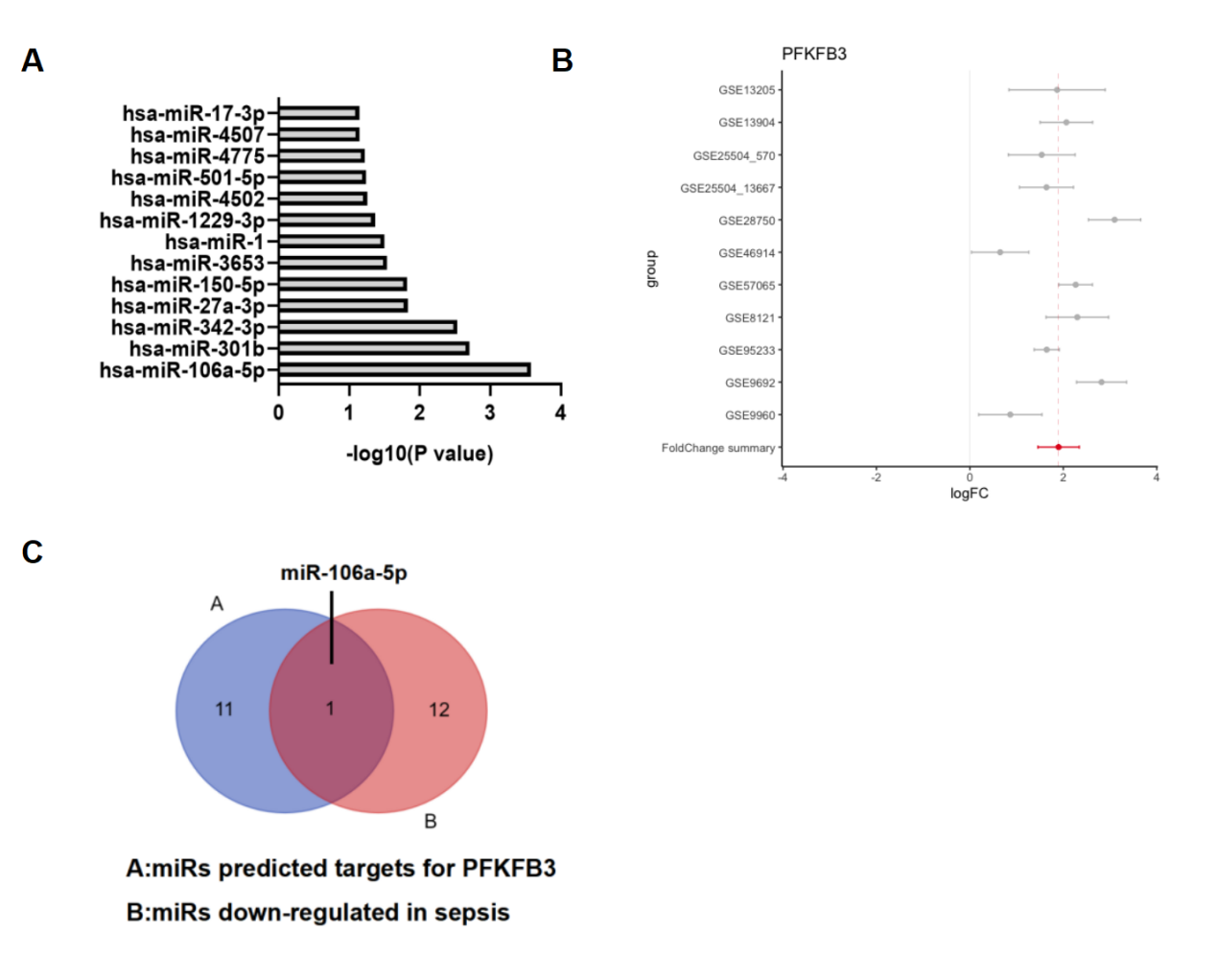


**Figure S2. (A)** The top 13 miRNAs significantly down-regulated in clinical sepsis samples. **(B)** The summary of differential expression of PFKFB3 in a series of GSE data. **(C)** Intersection analysis of miRNAs predicted targets for PFKFB3 and miRNAs down-regulated in sepsis.


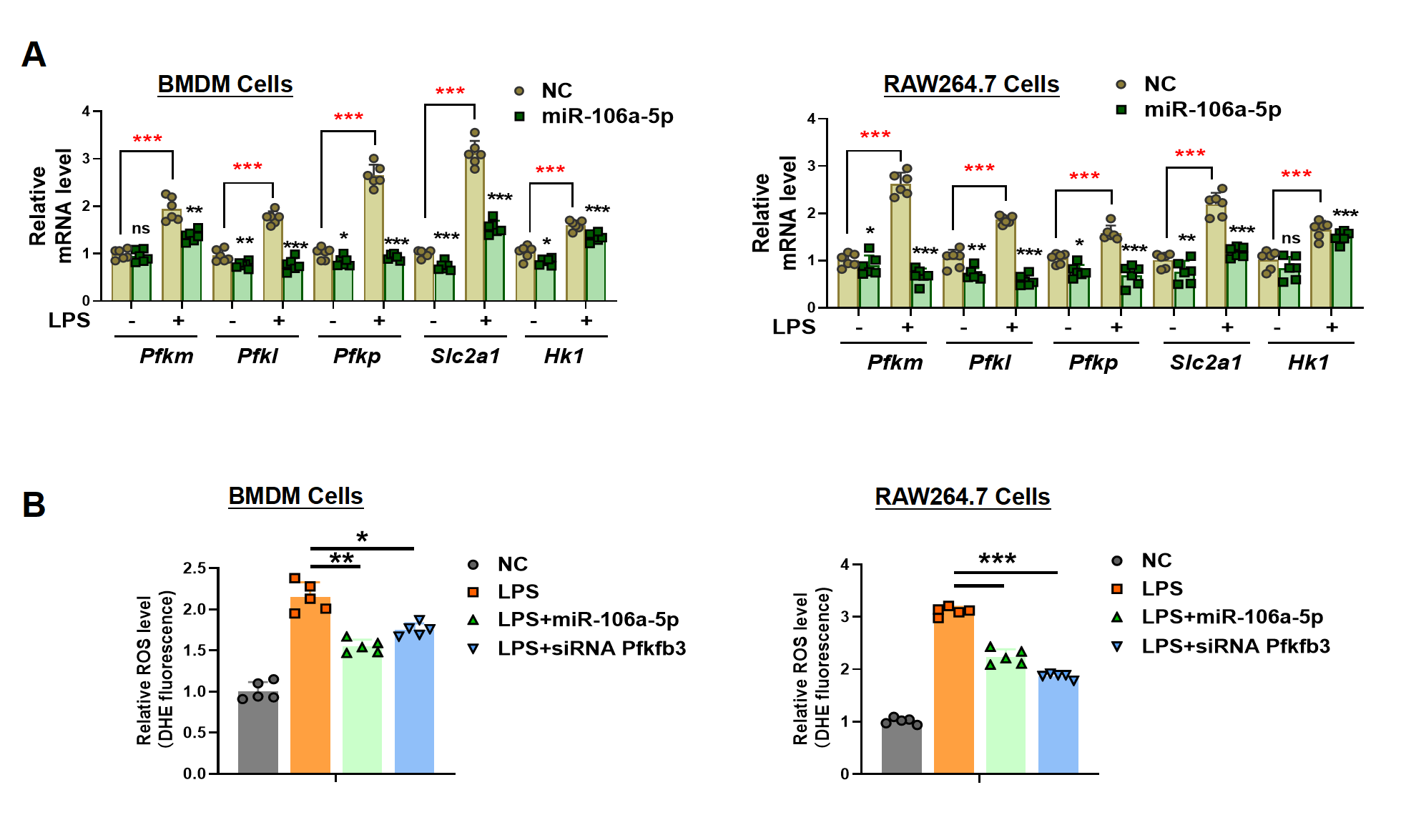


**Figure S3. (A)** The mRNA levels of glycolytic enzymes (*Pfkm, Pfkl, Pfkp,* and *Hk1*) and glucose transporter *Slc2a1* in LPS-treated BMDM and RAW264.7 cells with miR-106a-5p pretreated (n=6 per group). **(B)** Cellular ROS detection via DHE staining followed by flow cytometry in LPS-treated BMDM and RAW264.7 cells with miR-106a-5p mimic or si*Pfkfb3* pre-transfected (n=5 per group). Values are presented as means ± SD, *p<0.05, **p<0.01, ***p<0.001 (one-way ANOVA with Tukey’s *post hoc* test).


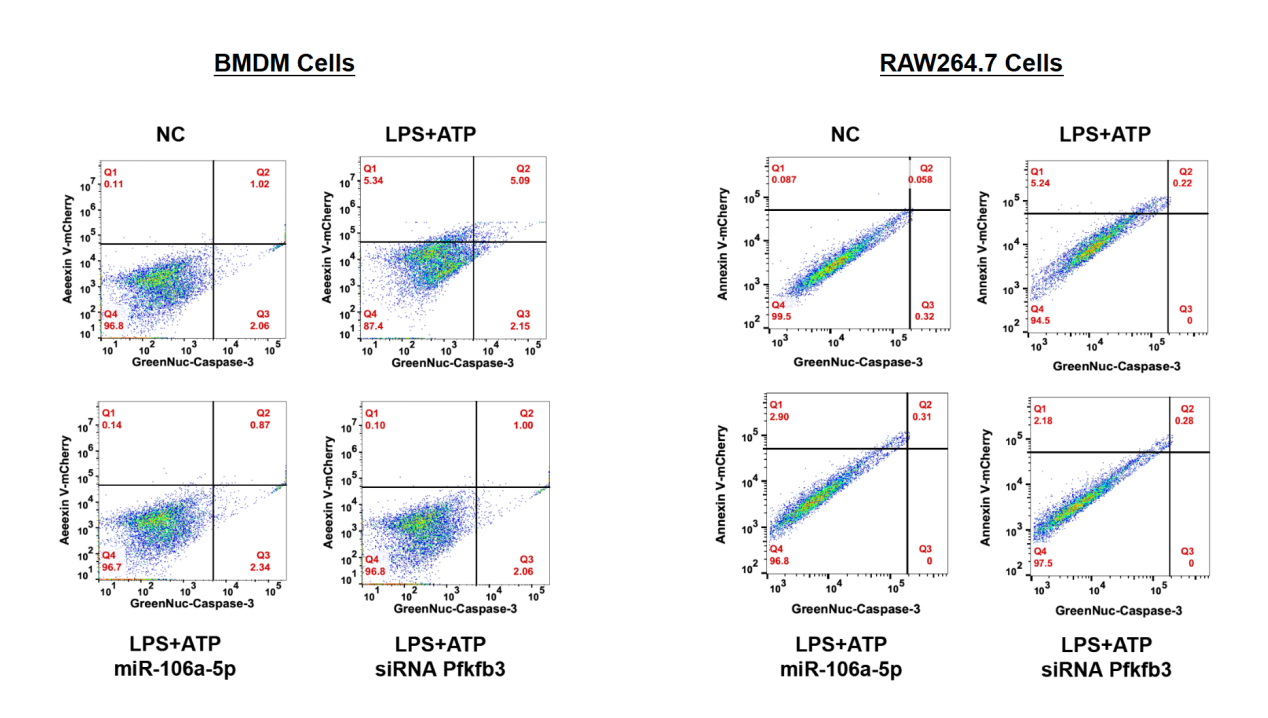


**Figure S4.** Flow cytometry analysis of Annexin V and Caspase 3 to demonstrate apoptosis or pyroptosis of miR-106a-5p mimic or si*Pfkfb3*-transfected BMDM and RAW264.7 cells challenged with vehicle or 100 ng/ml LPS for 24 h followed by incubating with ATP (4 mM) for 45 min (n=3 per group).


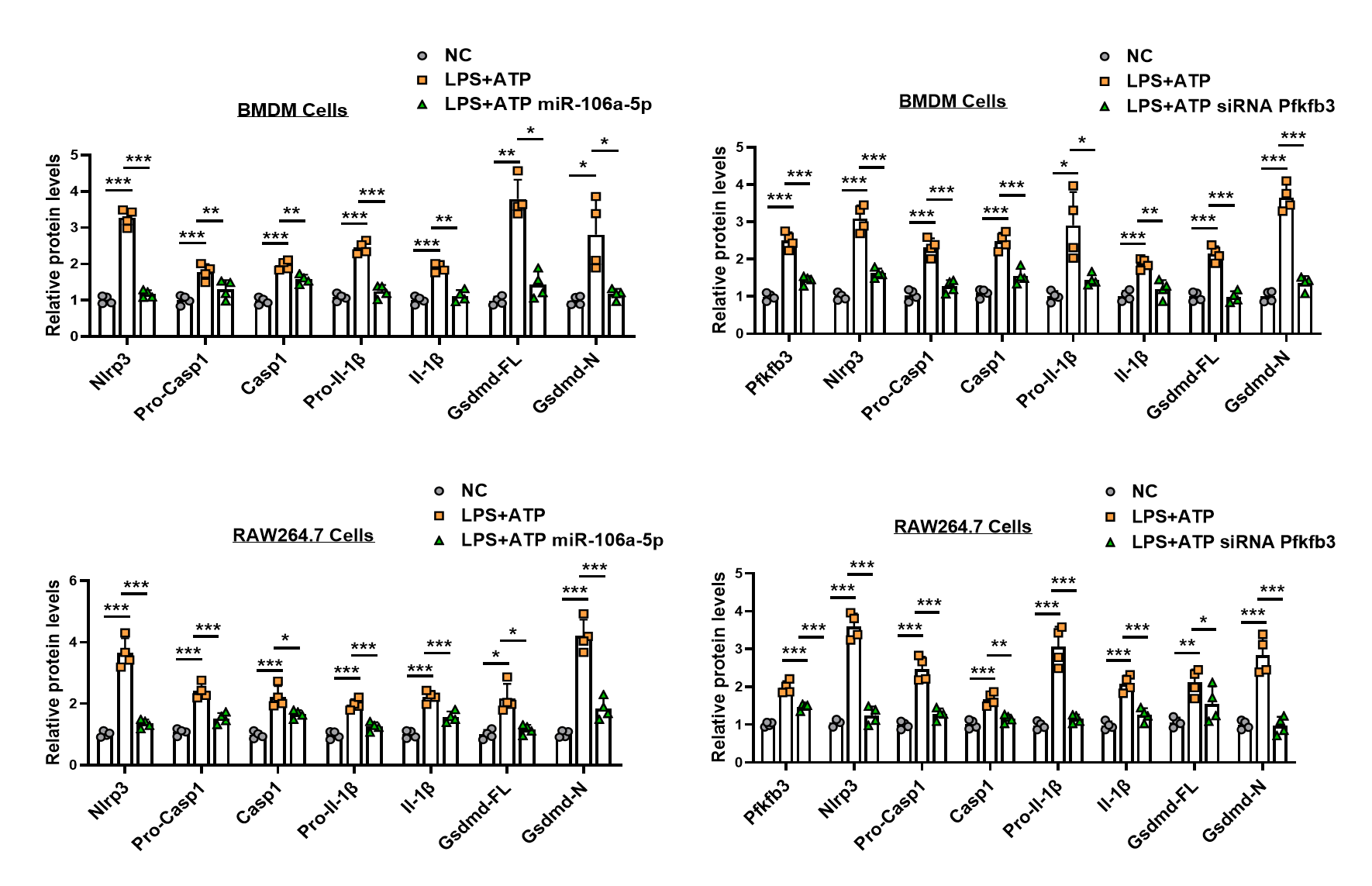


**Figure S5.** The quantification of Western blot images of of Pfkfb3, Nlrp3, Pro-Casp1, Cleaved Casp1, Pro-Il-1β, Cleaved Il-1β, full-length Gsdmd, and N-terminal fragment of Gsdmd in miR-106a-5p mimic or si*Pfkfb3*-transfected BMDM and RAW264.7 cells with Ctrl or LPS followed by 45 min ATP incubation (n=4 biological replicates).Values are presented as means ± SD, *p<0.05, **p<0.01, ***p<0.001 (one-way ANOVA with Tukey’s *post hoc* test).


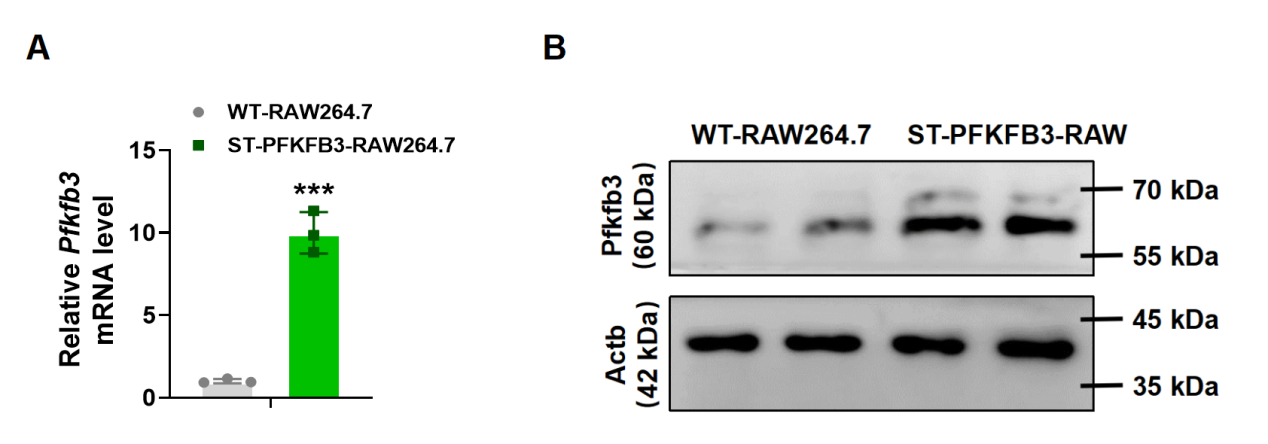


**Figure S6.** **(A)** The mRNA levels of *Pfkfb3* in WT-RAW264.7 and ST-Pfkfb3-RAW264.7 cells (n=3 per group). **(B)** The protein levels of Pfkfb3 in WT-RAW264.7 and ST-Pfkfb3-RAW264.7 cells (n=2 per group). Values are presented as means ± SD, ***p<0.001 (unpaired, two-tailed Student’s *t*-test).


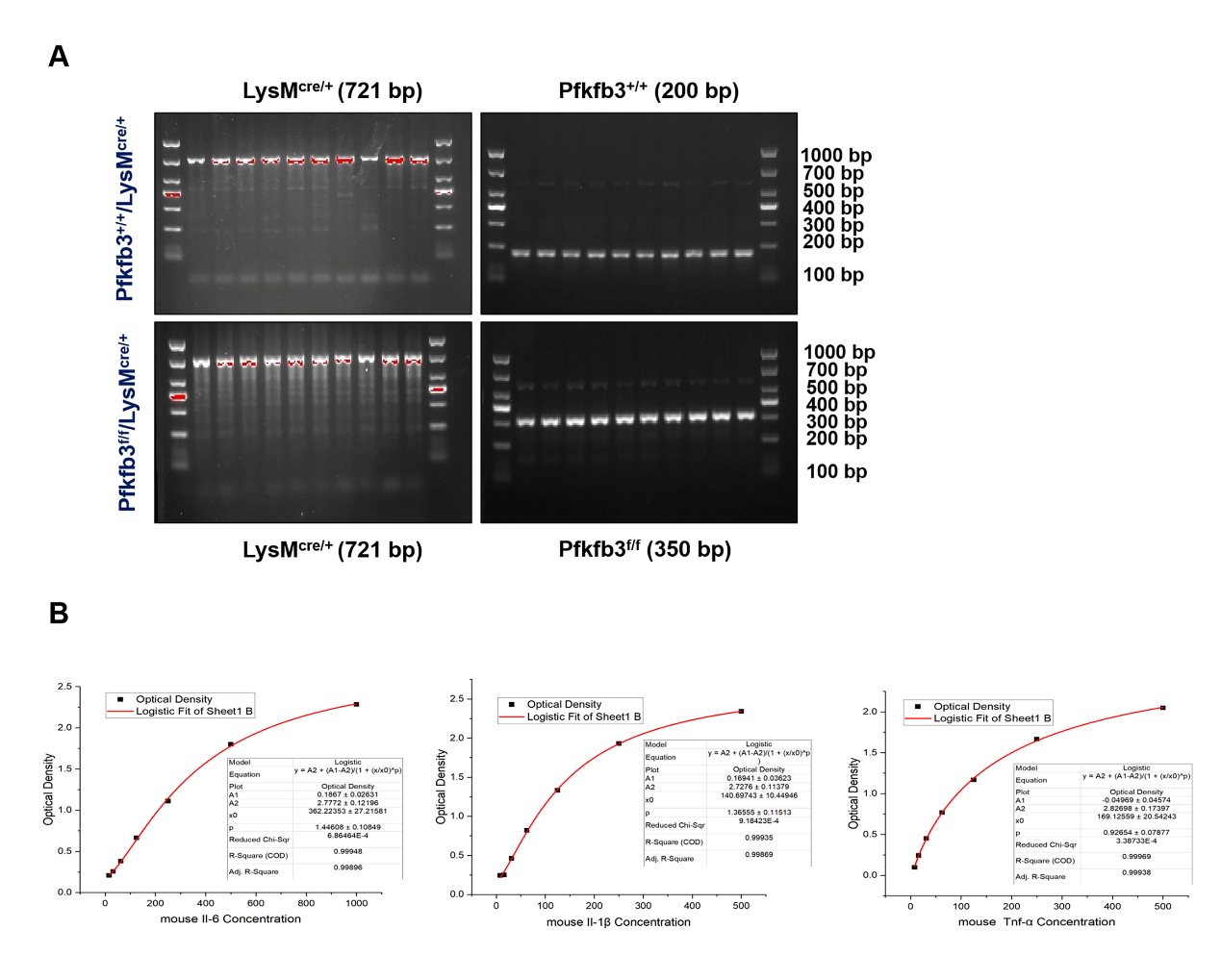


**Figure S7.** **(A)** Genotyping of *Pfkfb3*^+/+^/LysM^cre/+^ and *Pfkfb3*^flox/flox^/LysM^cre/+^ mice (n=10 mice per group). **(B)** Standard curve of ELISA analysis of Il-6, Il-1β and Tnf-α.

**Table S1. miRNAs that potentially target PFKFB3**

| **Human** | **Mouse** |
| --- | --- |
| 196-5p | 196-5p |
| 223-3p | 223-3p |
| 205-5p | 205-5p |
| 488-5p | 488-5p |
| 130-3p/301-3p/454-3p | 130-3p/301-3p |
| 19-3p | 19-3p |
| 17-5p/20-5p/93-5p/106a-5p/519-3p | 17-5p/20-5p/93-5p/106a-5p |
| 133a-3p.1 | 133a-3p.1 |
| 26-5p | 26-5p |
| 133a-3p/133b | 133-3p |

**Table S2. The summarization of selected Microarrays in GSE database.**

| **GSE** | **Sample** | **Platform** |
| --- | --- | --- |
| GSE13205 | 3 Ctrl vs 3 Sepsis | GPL20712 |
| GSE174507 | 6 Ctrl vs 12 Sepsis | GPL25134 |
| GSE101639 | 3 Ctrl vs 6 Sepsis | GPL18058 |
| GSE94717 | 3 Ctrl vs 12 Sepsis | GPL19449 |

| **GSE** | **Sample** | **Platform** |
| --- | --- | --- |
| GSE13205 | 8 Ctrl vs 13 Sepsis | GPL570 |
| GSE13904 | 18 Ctrl vs 52 Sepsis | GPL570 |
| GSE25504 | 71 Ctrl vs 63 Sepsis | GPL570 |
| GSE25504 | 71 Ctrl vs 63 Sepsis | GPL13667 |
| GSE28750 | 20 Ctrl vs 10 Sepsis | GPL570 |
| GSE46914 | 12 Ctrl vs 12 Sepsis | GPL570 |
| GSE57065 | 25 Ctrl vs 28 Sepsis | GPL570 |
| GSE8121 | 15 Ctrl vs 60 Sepsis | GPL570 |
| GSE95233 | 22 Ctrl vs 51 Sepsis | GPL570 |
| GSE9692 | 15 Ctrl vs 30 Sepsis | GPL570 |
| GSE9960 | 16 Ctrl vs 9 Sepsis | GPL570 |

**Table S3. Sequences of primers for quantitative RT-PCR**

| **Gene Name** | **Gene ID** | **Species Specificity** | **Sequences of Primers** |
| --- | --- | --- | --- |
| Actb | 11461 | Mouse | Forward 5’-GTGACGTTGACATCCGTAAAGA-3’ |
|  |  |  | Reverse 5’-GCCGGACTCATCGTACTCC-3’ |
| ACTB | 60 | Human | Forward 5’-CATGTACGTTGCTATCCAGGC-3’ |
|  |  |  | Reverse 5’-CTCCTTAATGTCACGCACGAT-3’ |
| Il-6 | 16193 | Mouse | Forward 5’-CTGCAAGAGACTTCCATCCAG-3’ |
|  |  |  | Reverse 5’-AGTGGTATAGACAGGTCTGTTGG-3’ |
| IL-6 | 3569 | Human | Forward 5’-ACTCACCTCTTCAGAACGAATTG-3’ |
|  |  |  | Reverse 5’-CCATCTTTGGAAGGTTCAGGTTG-3’ |
| Il-1β | 16176 | Mouse | Forward 5’-GAAATGCCACCTTTTGACAGTG-3’ |
|  |  |  | Reverse 5’-TGGATGCTCTCATCAGGACAG-3’ |
| IL-1β | 3553 | Human | Forward 5’-ATGATGGCTTATTACAGTGGCAA-3’ |
|  |  |  | Reverse 5’-GTCGGAGATTCGTAGCTGGA-3’ |
| Tnf-α | 21926 | Mouse | Forward 5’-CAGGCGGTGCCTATGTCTC-3’ |
|  |  |  | Reverse 5’-CGATCACCCCGAAGTTCAGTAG-3’ |
| TNF-α | 7124 | Human | Forward 5’-CCTCTCTCTAATCAGCCCTCTG-3’ |
|  |  |  | Reverse 5’-GAGGACCTGGGAGTAGATGAG-3’ |
| Nos2 | 18126 | Mouse | Forward 5’-GTTCTCAGCCCAACAATACAAGA-3’ |
|  |  |  | Reverse 5’-GTGGACGGGTCGATGTCAC-3’ |
| NOS2 | 4843 | Human | Forward 5’-TTCAGTATCACAACCTCAGCAAG-3’ |
|  |  |  | Reverse 5’-TGGACCTGCAAGTTAAAATCCC-3’ |
| Ccl-2 | 20296 | Mouse | Forward 5’-TAAAAACCTGGATCGGAACCAAA-3’ |
|  |  |  | Reverse 5’-GCATTAGCTTCAGATTTACGGGT-3’ |
| CCL-2 | 6347 | Human | Forward 5’-CAGCCAGATGCAATCAATGCC-3’ |
|  |  |  | Reverse 5’-TGGAATCCTGAACCCACTTCT-3’ |
| Pfkfb3 | 170768 | Mouse | Forward 5’-CAACTCCCCAACCGTGATTGT-3’ |
|  |  |  | Reverse 5’-TGAGGTAGCGAGTCAGCTTCT-3’ |
| PFKFB3 | 5209 | Human | Forward 5’-TTGGCGTCCCCACAAAAGT-3’ |
|  |  |  | Reverse 5’-AGTTGTAGGAGCTGTACTGCTT-3’ |
| Pfkm | 18642 | Mouse | Forward 5’-CATCGCCGTGTTGACCTCT-3’ |
|  |  |  | Reverse 5’-CCCGTGAAGATACCAACTCGG-3’ |
| Pfkl | 18641 | Mouse | Forward 5’-GGAGGCGAGAACATCAAGCC-3’ |
|  |  |  | Reverse 5’-GCACTGCCAATAATGGTGCC-3’ |
| Pfkp | 56421 | Mouse | Forward 5’-GGGACCATCATCGGTAGTGC-3’ |
|  |  |  | Reverse 5’-GTCCGCTCCACTCCTTTCG-3’ |
| Slc2a1 | 20525 | Mouse | Forward 5’-GCAGTTCGGCTATAACACTGG-3’ |
|  |  |  | Reverse 5’-GCGGTGGTTCCATGTTTGATTG-3’ |
| Hk1 | 15275 | Mouse | Forward 5’-AACGGCCTCCGTCAAGATG-3’ |
|  |  |  | Reverse 5’-GCCGAGATCCAGTGCAATG-3’ |

**Table S4. Sequences of primers for miRNA quantitative RT-PCR analysis**

| **Gene Name** | **Sequences of Primers** |
| --- | --- |
| MiRNA Stem-loop-Primer | 5’- GTCGTATCCAGTGCAGGGTCCGAGGTA  TTCGCACTGGATACGACCTACCT -3’ |
| U6 | Forward 5’-CTCGCTTCGGCAGCACA-3’ |
|  | Reverse 5’-AACGCTTCACGAATTTGCGT-3’ |
| MiR-106a-5p | Forward 5’- GCGCAAAGTGCTAACAGTGC-3’ |
|  | Reverse 5’- AGTGCAGGGTCCGAGGTATT-3’ |

**Table S5. Sequences of primers for PCR identification of Pfkfb3^+/+^/LysM^cre/+^ and Pfkfb3^flox/flox^/LysM^cre/+^ mice**

| **Name** | **Sequences of Primers** |
| --- | --- |
| LysM-KO | Forward 5'- CCCAGAAATGCCAGATTACG- 3' |
|  | Reverse 5'- CTTGGGCTGCCAGAATTT CTC-3' |
| Pfkfb3-KO | Forward 5’- GGAGTGGCCATTAGGTGGGGT T -3’ |
|  | Reverse 5’- CCAGCTTGGGCTACGCATTTAGTT -3’ |
